# Intelligent Automobile Bionic Cockpit Selection Considering Personalization Requirements: Multiple-Criterion Model and Decision-Making Method

**DOI:** 10.3390/biomimetics10100706

**Published:** 2025-10-17

**Authors:** Liangliang Shi, Shaolin Zhang, Tao Han, Niansong Liu, Guoquan Xie, Guangdong Tian

**Affiliations:** 1 State Key Laboratory of Intelligent Vehicle Safety Technology, China Automotive Engineering Research Institute Co., Ltd., Chongqing 401122, China; ll.shi@outlook.com (L.S.); liuniansong@caeri.com.cn (N.L.); 2 School of Mechanical Engineering, Shandong University, Jinan 250061, China; zsl10162022@mail.sdu.edu.cn (S.Z.); tao_han@mail.sdu.edu.cn (T.H.); 3 Key Laboratory of High Efficiency and Clean Mechanical Manufacture of Ministry of Education, Shandong University, Jinan 250061, China; 4 Department of Mechanical and Automation Engineering, The Chinese University of Hong Kong, Hong Kong, China; 5 Mechanical-Electrical and Vehicle Engineering, Beijing University of Civil Engineering and Architecture, Beijing 100044, China

**Keywords:** intelligent automobile bionic cockpits selection, decision-making, spherical fuzzy set, entropy measure

## Abstract

The extensive integration of intelligent and bionic technologies in the automotive industry has significantly heightened interest in the advancement of smart vehicle cockpits. The growing demand for automobile cockpit functions makes the personalization of intelligent automobile bionic cockpits more challenging. In addition, the evaluation and selection for cockpits considering multiple attributes remains incomplete, which hinders the development of intelligent automobile bionic cockpits. Thus, this paper constructed a multiple criterion model considering the personalization needs of drivers and passengers, which include comfort, security, and spiritual entertainment needs. A novel decision-making approach that merges the entropy measure and the Elimination and Choice Expressing Reality (ELECTRE) method is introduced to address the selection challenges of smart vehicle cockpits. This methodology incorporates the Spherical Fuzzy Set (SFS) to accurately gather and interpret the data within the decision matrix. This study employs a practical application by examining three types of intelligent automobile cockpits to validate the effectiveness of the proposed decision-making method. Through sensitivity analysis and comprehensive validation, the findings substantiate that the research offers a potent instrument for addressing the selection challenges associated with intelligent automobile cockpits, providing valuable insights for designers.

## 1. Introduction

As artificial intelligence, internet technologies, and communication technologies advance swiftly, intelligent automobiles have secured a crucial role and emerged as the most extensively utilized mode of transportation in contemporary society. In addition, the demands of drivers and passengers for cockpits are not limited to safety features [1,2]. The innovative concept of intelligent automobile bionic cockpits is proposed, which integrates advanced technologies to provide an enhanced driving experience. Its development can be attributed to the growing demand for connected and personalized driving experiences [3,4]. In recent years, numerous scholars have investigated the incorporation of diverse technologies, including the Internet of Things (IoT), Machine Learning (ML), Bionics Design (BD), and Artificial Intelligence (AI) aiming to enhance the comfort and functionality of cockpit [5]. Especially during the design process, the introduction of BD technology has played a crucial role in upgrading and improving the comprehensive performance of the intelligent cockpit. However, as drivers and passengers demand a richer in-vehicle environment, the development of more personalized intelligent automobile bionics cockpits is promising.

The vehicle cockpit is originally designed to separate the driver from the outside environment, creating a relatively safe driving environment [6]. As technology advances and the demand for various services grows, consumers are becoming increasingly attentive to their experiences within the cockpits. In addition to security, comfort in automobile cockpits becomes a new goal for drivers [7,8]. Human–machine interaction, as an emerging cockpit comfort evaluation index, can effectively improve the driving feeling and feed into security to some extent, which has been viewed as the research hot-point of cockpit comfort [9,10]. In addition, the spiritual entertainment needs of drivers gradually attract attention, which is also viewed as an important criterion for evaluating the intelligent cockpit. However, recent studies have primarily concentrated on cockpit comfort, leaving significant research gaps in other areas. To better describe the personalized needs of the driver in the cockpit from a general perspective and provide references for future studies for intelligent automobile cockpits, it is crucial to construct a systemic multiple-criterion model.

With the emergence of multiple intelligent vehicles and intelligent automobile cockpits, it is important to rationally evaluate and select the optimal intelligent automobile cockpits for satisfying personalized needs. Previous studies have worked on the evaluation of comfort, color effects, and passenger experiences in intelligent automobile cockpits [11,12]. The intelligent automobile cockpit evaluation based on multiple personalized needs remains a large gap [13]. In addition, few studies have conducted comparative analyses of different types of intelligent automobile cockpits, which limits their practical applicability. In this context, multi-criteria decision-making (MCDM) methods have been widely adopted in various fields, including programming language selection [14], Internet of Things (IoT) solutions [15], complex engineering and data-driven research [16], green material selection [17], intelligent transportation and safety design optimization [18,19], and remanufacturing system decision-making [20,21], due to their proven effectiveness in handling multiple evaluation dimensions and uncertain information [22]. These methods thus provide a suitable framework for systematically assessing intelligent automobile cockpits. Therefore, to meet diverse personalized needs and ensure practical relevance, it is essential to introduce a multi-criterion decision-making approach that considers multiple evaluation indices and case comparisons.

Focusing on the abovementioned problems, this paper delves into the multi-criterion model and decision-making approach for selecting intelligent automobile bionic cockpits. It distinguishes itself from prior work through several key contributions: (1) A multi-level framework encompassing 25 indicators is established in this study, comprehensively covering safety, comfort, and spiritual or entertainment requirements, realistically reflecting the complex and personalized conflicts of needs experienced in real-world scenarios; (2) A hybrid decision-making approach is developed by integrating the entropy measure with the Elimination and Choice Expressing Reality (ELECTRE) method within spherical fuzzy sets, thereby effectively overcoming the shortcomings of existing methods in processing expert judgments characterized by high conflict and hesitation; (3) Three representative intelligent automobile bionic cockpits are evaluated, with results demonstrating that the proposed method can serve as a reliable and scientifically sound tool for the optimal selection of intelligent automobile bionic cockpits.

The structure of the subsequent sections of this study is organized in the following manner: Section 2 provides a literature review and discusses the multiple criterion model. Section 3 proposes a fuzzy multi-criterion decision-making model that combines the fuzzy entropy method with the fuzzy ELECTRE method. Section 4 presents a case study on intelligent automobile bionic cockpits to demonstrate the application of the proposed method. Section 5 conducts the sensitivity analysis and validation. Section 6 provides the conclusion of the study.

## 2. Multiple-Criterion Evaluation Model for Intelligent Automobile Bionic Cockpit

### 2.1. Literature Review

Automobiles hold a pivotal role in daily life, and with ongoing advancements in artificial intelligence, internet, and communication technologies, the presence of intelligent cars on the roads is escalating rapidly [23]. Many companies are advancing the development of more intelligent vehicles, utilizing cutting-edge technologies and innovative materials to cater to the unique demands people have for intelligent cars [24].

Park et al. [25] highlight a shift in consumer focus over time from the underlying technology of intelligent cars to a greater emphasis on cockpit comfort and the driving experience. Intelligent vehicles offer numerous advantages such as enhanced safety, reduced need for infrastructure investment, better fuel efficiency, and decreased traffic congestion. However, the paramount benefit lies in the increased relaxation and comfort for passengers. Consequently, automobile manufacturers are increasingly dedicating efforts to enhance cockpit comfort. Beggiato et al. [26] emphasize the critical role of comfort in the widespread acceptance of intelligent cars. Research indicates that for products like intelligent vehicles, consumer satisfaction is a crucial element in their success. Consequently, ensuring comfort for users emerges as a fundamental objective across various industries. This is underpinned by the principle that the core of intelligence lies in facilitating user convenience and comfort. Cockpit comfort is one of the important factors in the evaluation of intelligent cockpits. Indicators for evaluating comfort include seat comfort, the efficiency of air conditioning and heating systems, noise and vibration levels, and the atmosphere in the cockpit. These indicators can be evaluated through objective tests and subjective questionnaires.

For drivers, comfort encompasses not just satisfaction derived from the driving experience but also pertains to driving safety and the long-term health implications for the driver [27]. Similarly, Sharma et al. [28] point out that for passengers, comfort is a paramount consideration, as it impacts health and productivity. In the contemporary automotive landscape, the enhancement of driving safety and comfort remains a persistent focus in vehicle design [29]. Passenger comfort analysis and the evaluation process plays a crucial role. Human comfort, a subjective sensation, is intricately multifaceted, encompassing aspects such as optical environment comfort, acoustic, and thermal. As Arvidsson et al. [30] discusses, acoustic comfort has a significant impact on passengers by influencing their stress levels, susceptibility to distraction, and cognitive performance. Furthermore, studies on thermal comfort, such as those by Xue et al. [31], have shown that the thermal environment impacts physiological and behavioral patterns, thereby influencing work efficiency outdoors. The optical environment, serving as a primary cue for the human biological clock, has diverse effects on sleep [32], mood [33], alertness [34] and so on, underlining the complexity and comprehensive nature of human comfort. Factors affecting personal comfort have the same impact on driver and passenger comfort in the automotive cockpit. In the assessment of cockpit comfort, the majority of existing research has concentrated on physical factors that influence comfort, such as seating position, vibration, and noise. However, few scholars have explored the use of physiological signals to gauge the comfort level within intelligent car cockpits, as highlighted by Su and Jia [35]. In evaluations of indoor human comfort, the acoustic environment, lighting environment, and thermal environment are recognized as the three primary physical factors that impact human comfort. Overall, methodologies for assessing passenger comfort in the context of intelligent cockpits are relatively scarce. In particular, there is a notable gap in research focusing on intelligent cars from the perspective of customer requirements, as identified by Park et al. [25].

Furthermore, the car cockpit is evolving into a room-like interior environment. According to Sun et al. [36], future advancements in intelligent cockpits will allow passengers to disengage from driving tasks, freeing up time for recreational activities and pursuits beyond driving. In their research on passenger behavior within the cockpit, some studies observed that participants commonly brought everyday items like paper documents and computing devices for use during non-driving periods. The prevalent activities identified included reading, engaging in social networking on mobile devices, browsing the internet, and watching videos on laptops or iPads, indicating a diverse range of leisure pursuits undertaken by passengers when not involved in driving [37]. Users have high expectations of self-driving cars because they currently do not have time for other recreational and spiritual activities in a traditional manually driven vehicle and such non-driving related activities have many possibilities for development. Although the cockpit entertainment segment represents a relatively nascent area within the automotive industry, there is a paucity of research regarding passenger acceptance and preference for these advancements. However, as a trend in intelligent cockpits, entertainment systems are also bound to be an important comfort factor, so the development of intelligent cockpits at this stage should also take into account the spiritual and entertainment requirements of passengers. Indeed, as expectations evolve, there will be an increasing demand for enhanced spiritual and entertainment options within the car cockpit. Catering to these elevated desires should become a pivotal consideration in the development of intelligent cockpit functionalities.

In recent years, MCDM methods have been widely applied. Common methods include AHP [38], TOPSIS [39], VIKOR [40], PROMETHEE [41], and ELECTRE [42]. AHP offers clear hierarchical structuring and facilitates subjective weighting, but it may suffer from inconsistencies due to heavy reliance on expert judgment. TOPSIS provides intuitive ranking based on proximity to an ideal solution, though it handles uncertainty poorly. VIKOR balances conflicting criteria through compromise solutions but may overlook user-specific preferences. PROMETHEE enables flexible pairwise comparisons and detailed ranking but is sensitive to the choice of preference function. ELECTRE effectively addresses incompleteness and uncertainty, yet alone it may not fully capture user hesitancy or fuzzy perceptions. While each method has strengths, their limitations make them insufficient for evaluating complex, personalized requirements in intelligent automobile cockpits.

In addition to MCDM methods, various fuzzy set approaches have been widely applied to handle uncertainty and vagueness in decision-making. Traditional fuzzy sets (FS) [43] allow modeling of partial membership but cannot fully capture hesitation. Intuitionistic fuzzy sets (IFS) [44] introduce a degree of non-membership alongside membership, providing more flexibility, while interval-valued intuitionistic fuzzy sets (IVIFS) [45] further accommodate ranges of uncertainty. However, these methods may still struggle to comprehensively represent the simultaneous positive, neutral, and negative preferences that experts may express in complex, personalized evaluations.

To address these limitations, this study adopts spherical fuzzy sets (SFS). An SFS extends traditional fuzzy frameworks by incorporating positive, neutral, and negative membership degrees along with a refusal degree, effectively capturing hesitancy, uncertainty, and nuanced expert judgments. This makes SFS particularly suitable for evaluating intelligent automobile bionic cockpits, where passenger preferences are multifaceted and subjective. Combining the ELECTRE method with spherical fuzzy sets (SFS) enables more accurate and robust multi-criteria decision-making under uncertainty, effectively handling hesitancy and subjective, user-centered evaluations, and outperforming conventional fuzzy approaches in this context.

The literature review reveals a noticeable gap in models for a comprehensive evaluation that fully encompasses the holistic personalized requirements of automobile cockpits. When assessing the personalized needs of intelligent vehicle cockpits, the scientific and thorough selection of evaluation indicators critically influences the accuracy and objectivity of the results. Hence, a multi-faceted comprehensive evaluation approach is essential to thoroughly assess the personalized requirements of intelligent automobile cockpits, considering various aspects like security, comfort, and spiritual entertainment needs. With the advancement of intelligent vehicles and the subsequent redesign and reorganization of the intelligent cockpit, enhancing the passenger experience has become paramount. This underscores the growing necessity for a comprehensive evaluation of the personalized requirements of the intelligent cockpit.

### 2.2. Multiple-Criterion Evaluation Model

**Figure 1:**
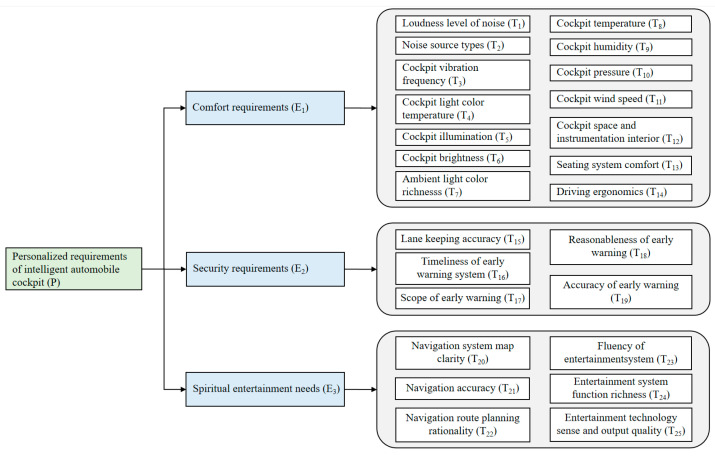
Comprehensive evaluation index system for the impact of intelligent automobile bionic cockpit personalized requirements.

Assessing the impact of personalized requirements on an intelligent automobile bionic cockpit system can be challenging due to the need for a balanced number of indicators. Too few indicators can lead to an incomplete evaluation of the system, while too many can make the evaluation process overly complex. To address this, a comprehensive set of 25 secondary indicators has been defined to assess passengers’ personalized requirements in the cockpit. These indicators are organized under three influencing factors: safety, comfort, and spiritual or entertainment requirements, as shown in Figure 1.

In this study, the comfort dimension is considered a key factor for passenger satisfaction and overall experience. It is segmented into four subordinate systems: light environment, sound environment, thermal environment, and human–computer interaction. These systems directly influence passengers’ physical and psychological comfort, shaping how they perceive the in-cabin environment. Sound environment affects auditory perception and stress levels, which are critical for long-duration trips and overall well-being. Three-level indicators include loudness level of noise (T_1_), noise source types (T_2_), and cockpit vibration frequency (T_3_). Light environment governs visibility, eye strain, and overall cabin atmosphere, impacting mood, alertness, and driving performance. Three-level indicators include: cockpit light color temperature (T_4_), cockpit illumination (T_5_), cockpit brightness (T_6_), and ambient light color richness (T_7_). Thermal environment determines physical comfort and contributes to passengers’ physiological satisfaction. Three-level indicators include: cockpit temperature (T_8_), cockpit humidity (T_9_), cockpit pressure (T_10_), and cockpit wind speed (T_11_). Human–computer interaction is essential for usability and ergonomic well-being, directly affecting ease of control and passenger satisfaction. Three-level indicators include: cockpit space and instrumentation interior (T_12_), seating system comfort (T_13_), and driving ergonomics (T_14_).

Safety is the most critical dimension, as it directly safeguards passengers’ lives and ensures the overall reliability of the vehicle. To maintain a secure driving environment and respond promptly to potential hazards, the safety requirements are divided into two secondary indicators: lane keeping and early warning systems. The three-level indicators of the lane-keeping system include lane keeping accuracy (T_15_), which measures the vehicle’s ability to stay within lanes, directly impacting accident prevention. The tertiary indicators for evaluating the early warning system encompass the timeliness of the scope of early warning system (T_16_), the scope of early warning (T_17_), the reasonableness of early warning (T_18_), and the accuracy of the early warning (T_19_), reflecting the system’s effectiveness in alerting drivers and mitigating risks.

Spiritual and recreational requirements are essential for enhancing passengers’ in-cabin experience, supporting cognitive engagement, entertainment, and overall satisfaction. These requirements are divided into two secondary indicators: navigation system and multimedia entertainment system. The three-level indicators of the navigation system map clarity (T_20_), navigation accuracy (T_21_), and navigation route planning rationality (T_22_), ensuring passengers can travel efficiently and confidently. The three-level indicators of the multimedia entertainment system include fluency of entertainment system (T_23_), entertainment system function richness (T_24_), entertainment technology sense and output quality (T_25_), reflecting both performance and the overall user experience.

To ensure that the evaluation indicators are not only comprehensive but also measurable, this study provides standardized definitions for those indicators that have been refined into detailed sub-indicators.

For the comfort criteria, in the sound environment system, loudness level of noise (T_1_) is measured in decibels using a sound level meter. Noise source types (T_2_) classify the origins of noise, which can be identified and quantified via acoustic analysis and frequency spectrum measurement. Cockpit vibration frequency (T_3_) can be quantified using accelerometers or vibration sensors. In the light environment system, cockpit light color temperature (T_4_) refers to the color range of cockpit lighting, which can be quantified in Kelvin using a colorimeter. Cockpit illumination (T_5_) indicates brightness level, which can be measured in lux using a lux meter. Cockpit brightness (T_6_) describes the perceived intensity of lighting, which can be quantified via photometric measurement or subjective brightness rating scales. Ambient light color richness (T_7_) reflects the diversity and variation in ambient light colors, which can be measured with a spectrometer or colorimetry analysis. In the thermal environment system, cockpit temperature (T_8_), cockpit humidity (T_9_), cockpit pressure (T_10_), and cockpit wind speed (T_11_) correspond to directly measurable environmental parameters, which can be quantified using instruments or sensors.

Within the human–computer interaction system, cockpit space and instrumentation interior (T_12_) refers to ergonomic layout and accessibility, which can be quantified by measuring reach distances and available clearance for controls. Seating system comfort (T_13_) denotes support and adjustability of seats, which can be quantified by assessing seat pressure distribution and adjustable range. Driving ergonomics (T_14_) evaluates posture adaptability during driving, which can be quantified by measuring joint angles and postural deviations using motion capture or posture sensors.

In terms of safety, lane keeping accuracy (T_15_) can be quantified by measuring the vehicle’s deviation from lane markings using sensors or camera systems. The early warning system is evaluated through four tertiary indicators: Timeliness of the early warning system (T_16_) can be quantified by recording the response time from hazard detection to alert issuance. Scope of early warning (T_17_) can be measured by the range of hazardous areas the system can monitor. Reasonableness of early warning (T_18_) can be assessed by comparing issued alerts with actual risk events, using system logs and event records. Accuracy of the early warning (T_19_) can be quantified by calculating the proportion of correct alerts to total alerts.

For spiritual and recreational needs, the indicators cover navigation and multimedia entertainment systems. Navigation clarity (T_20_) and navigation accuracy (T_21_) are measured by map legibility and GPS deviation, while route planning rationality (T_22_) is measured by comparison with optimal route criteria. The multimedia system is assessed by fluency (T_23_), functional richness (T_24_), technological sense and output quality (T_25_), measured via system response time, number of features, expert scores, and audio–visual fidelity metrics, respectively. These definitions draw on commonly accepted engineering practices and human–machine interaction standards, which help ensure greater clarity and objectivity in the evaluation framework.

## 3. Solution Method

### 3.1. Spherical Fuzzy Theory

**Definition 1.**  *The concept of a spherical fuzzy set U within the universe Y is defined as U = {(y, a_u_(y), b_u_(y), c_u_(y))|y ∈ Y}, where c_u_, b_u_, a_u_ represents the degree of negative membership, neutral membership, and positive membership for each element y in Y. These membership degrees are confined within the interval* [0, 1] *and are subject to a bounding condition* $a_{u}^{2}+b_{u}^{2}+c_{u}^{2}\leq 1,\forall y\in Y$ *that ensures the consistency of the set. Additionally, the definition of refusal degree e_u_ is* $e_{u}(y)=\sqrt{1-a_{u}^{2}-b_{u}^{2}-c_{u}^{2}}$ *. For convenience, the triplet (a_u_, b_u_, c_u_) is labeled as the spherical fuzzy number (SFN).*

**Definition 2** (**[46]**)**.**  *For the SFN U = (a_u_, b_u_, c_u_), the score degree is defined as follows:*

(1) $$S(u)={\left(2a_{u}-\frac{b_{u}}{2}\right)}^{2}-{\left(c_{u}-\frac{b_{u}}{2}\right)}^{2}$$

**Definition 3** (**[47]**)**.**  *For the SFN U = (a_u_, b_u_, c_u_), the accuracy degree is defined as follows:*

(2) $$A(u)=a_{u}^{2}+b_{u}^{2}+c_{u}^{2}$$

**Definition 4** (**[48]**)**.**  *For the comparison of two SFNs U*_1_*= a*_1_*, b*_2_*, c*_3_*, U*_2_*= a*_2_*, b*_2_*, c*_2_.

If $S(u_{1})>S(u_{2})$ then $u_{1}>u_{2}$

If $S(u_{1})=S(u_{2})$ then (1) if $A(u_{1})>A(u_{2})$ then $u_{1}>u_{2}$. (2) if $A(u_{1})=A(u_{2})$ then $u_{1}\sim u_{2}$.

**Definition 5** (**[48]**)**.**  *For three SFNs U = (a_u_, b_u_, c_u_), U*_1_*= a*_1_*, b*_2_*, c*_3_ *and U*_2_*= a*_2_*, b*_2_*, c*_2_*, the elementary operations are defined as follows:*

$$1.u_{1}⊕u_{2}=(\sqrt{a_{1}^{2}+a_{2}^{2}+a_{1}^{2}a_{2}^{2}},\sqrt{{\left(1-a_{2}^{2}\right)}^{2}b_{1}^{2}+{\left(1-a_{1}^{2}\right)}^{2}b_{2}^{2}-b_{1}^{2}b_{2}^{2}},c_{1}c_{2})$$

$$2.u_{1}⊗u_{2}=(a_{1}a_{2},\sqrt{{\left(1-c_{2}\right)}^{2}b_{1}^{2}+{\left(1-c_{1}\right)}^{2}b_{2}^{2}-b_{1}^{2}b_{2}^{2}},\sqrt{c_{1}^{2}+c_{2}^{2}-c_{1}^{2}c_{2}^{2}})$$

$$3.\lambda u\;\;=(\sqrt{1-{\left(1-a^{2}\right)}^{\lambda }},\sqrt{\left({\left(1-a^{2}\right)}^{\lambda }-{\left(1-a^{2}-b^{2}\right)}^{\lambda }\right)},c^{\lambda }),\lambda >0$$

$$4.u^{\lambda }=(a^{\lambda },\sqrt{\left({\left(1-c^{2}\right)}^{\lambda }-{\left(1-c^{2}-b^{2}\right)}^{\lambda }\right)},\sqrt{1-{\left(1-c^{2}\right)}^{\lambda }}),\lambda >0$$

**Definition 6** (**[48]**)**.**  *For a finite collection U_t_ = (a_t_, b_t_, c_t_) (t =* 1*,* 2*,* 3*,…, m) of SFNs, based on the normalized weight vector* $k=k_{1},k_{2},\ldots ,k_{m}$*, the spherical weighted arithmetic mean (SWAM) operator is defined as follows:*

(3) $$SWAM_{k}(u_{1},u_{2},\ldots ,u_{m})=k_{1}u_{1}⊕k_{2}u_{2}⊕\cdots ⊕k_{m}u_{m}=(\sqrt{1-\prod_{t=1}^{m}{\left(1-a_{t}^{2}\right)}^{k_{t}}},\sqrt{\prod_{t=1}^{m}{\left(1-a_{t}^{2}\right)}^{k_{t}}-\prod_{t=1}^{m}{\left(1-a_{t}^{2}-b_{t}^{2}\right)}^{k_{t}}},\prod_{t=1}^{m}c_{t}^{k_{t}})$$

### 3.2. Spherical Fuzzy Entropy Method

In the fuzzy environment, how to measure uncertain information is a difficult issue. The entropy measure, first proposed by Zadeh, has been used as a useful mathematical tool to describe the fuzzy degree [43]. A lot of fuzzy sets have been incorporated into the entropy measure to solve uncertain problems in a wide range of industries [49]. In the decision-making process, the entropy measure is commonly used to obtain the criterion weights, for it can acquire the information directly from the decision matrix without additional information from experts, which can reduce the subjectivity to some extent [50]. In this study, an entropy measure combined with the spherical fuzzy set, which is spherical fuzzy entropy, is introduced to obtain the criterion weights. The specific definitions are shown as follows.

For two SFNs *M* = (*a_m_*(*y*), *b_m_*(*y*), *c_m_*(*y*)) and *N* = (*a_n_*(*y*), *b_n_*(*y*), *c_n_*(*y*)) on *Y*, a real value function *E* can be defined as the entropy measure for SFSs (SFS ∈ [0, 1]) when the following properties are satisfied:

(1) If *M* is a crisp set, *E*(*M*) = 0;
(2) If *a_M_* (*y*) = *c_M_*(*y*) and *b_M_*(*y*) = 0.5 for all *y* ∈ *Y*, *E*(*M*) = 1;
(3) *E*(*M*) = *E*(*M^c^*);
(4) If *b*^2^*_M_*(*y*) ≤ *b*^2^*_N_*(*y*) and *a*^2^*_M_*(*y*) ≤ *a*^2^*_N_*(*y*) ≤ *c*^2^*_N_*(*y*) ≤ *c*^2^*_M_*(*y*) or *c*^2^*_M_*(*y*) ≤ *c*^2^*_N_*(*y*) ≤ *a*^2^*_N_*(*y*) ≤ *a*^2^*_M_*(*y*) for all *y* ∈ *Y*, *E*(*M*) ≤ *E*(*N*).

Then, the SFS-Entropy measure can be expressed as

(4) $$E(M)=\frac{1}{m}\sum_{i=1}^{m}\left(1-\frac{4}{5}\left[\left|a_{M}^{2}(y_{i})-c_{M}^{2}(y_{i})\right|+\left|b_{M}^{2}(y_{i})-0.25\right|\right]\right)$$

Based on the defined SFS-Entropy measure concept, the criterion weights can be computed as following steps:

Step 1. Constructed the decision matrix in the form of SFNs, as shown in Equation (5).

(5) $${ℵ}_{H_{t}}=\begin{array}{c} Z_{1}\\ Z_{2}\\ \vdots \\ Z_{p} \end{array}\begin{array}{cccccc} & T_{1} & T_{2} & \ldots & T_{p} & \\ ( & \begin{array}{c} \left(a_{11}^{\left(t\right)},b_{11}^{\left(t\right)},c_{11}^{\left(t\right)}\right)\\ \left(a_{21}^{\left(t\right)},b_{21}^{\left(t\right)},c_{21}^{\left(t\right)}\right)\\ \vdots \\ \left(a_{p1}^{\left(t\right)},b_{p1}^{\left(t\right)},c_{p1}^{\left(t\right)}\right) \end{array} & \begin{array}{c} \left(a_{12}^{\left(t\right)},b_{12}^{\left(t\right)},c_{12}^{\left(t\right)}\right)\\ \left(a_{22}^{\left(t\right)},b_{22}^{\left(t\right)},c_{22}^{\left(t\right)}\right)\\ \vdots \\ \left(a_{p2}^{\left(t\right)},b_{p2}^{\left(t\right)},c_{p2}^{\left(t\right)}\right) \end{array} & \begin{array}{c} \ldots \\ \ldots \\ \ddots \\ \ldots \end{array} & \begin{array}{c} \left(a_{2q}^{\left(t\right)},b_{2q}^{\left(t\right)},c_{2q}^{\left(t\right)}\right)\\ \left(a_{2q}^{\left(t\right)},b_{2q}^{\left(t\right)},c_{2q}^{\left(t\right)}\right)\\ \vdots \\ \left(a_{pq}^{\left(t\right)},b_{pq}^{\left(t\right)},c_{pq}^{\left(t\right)}\right) \end{array} & ) \end{array}$$

Step 2. Compute the SFS-Entropy measure of each criterion using Equation (6).

(6) $$e_{j}=\frac{1}{m}\sum_{i=1}^{m}\left(1-\frac{4}{5}\left[\left|a_{ij}^{2}-c_{ij}^{2}\right|+\left|b_{ij}^{2}-0.25\right|\right]\right)$$

Step 3. Obtain the criterion weights using Equation (7).

(7) $$w_{j}=\frac{1-e_{j}}{\sum_{j=1}^{n}\left(1-e_{j}\right)}$$

### 3.3. Fuzzy ELECTRE Method

This section is devoted to presenting an important method for solving MCGDM problems with incomplete information that is based on ELECTRE approaches [51]. The main goal of the proposed method is to collect confusing SFN data to produce more real-world and accurate findings. Further, to clarify the relationship between and differentiation between these strategies, a combined flowchart diagram of both approaches is shown. Any MCGDM problem’s mathematical description needs to be clarified for understanding before the entire technique is outlined [52].

The foundation of each MCGDM model consists of three core elements: alternatives, criteria, and decision-making experts. The principal goal of any decision-making method is to identify the most suitable alternative from the set *Z* = {*Z*_1_, *Z*_1_,…, *Z_p_*}. To oversee the various phases and dimensions of the decision-making process, a group of *m* decision-makers *H*_1_, *H*_2_,…, *H_m_* is invited to contribute their subjective expertise. To ensure the reliability and validity of the evaluations, the experts were selected based on specific criteria. They possess professional knowledge in intelligent automobile design, human–machine interaction, bionics, vehicle ergonomics, and intelligent cockpit design, and all have at least five years of experience in automotive or related fields. Selection criteria included familiarity with cockpit design and evaluation methods, prior involvement in research or practical projects related to intelligent vehicle systems, and the ability to provide informed judgments across multiple evaluation dimensions. The extent of each expert’s participation is quantified by a weight vector $k=k_{1},k_{2},\ldots ,k_{m}$. The efficacy of the alternatives is evaluated by *q* risk factors *T*_1_, *T*_2_,…, *T_q_*, each significantly impacting the performance of the alternatives. To address such issues, the flexible techniques of Spherical Fuzzy ELECTRE (SF-ELECTRE) are delineated in the subsequent steps:

Step 1: Construction of the spherical fuzzy decision matrix.

To reach a well-informed conclusion, experienced and knowledgeable decision-makers conduct an exhaustive analysis of risk factors, precedence, and the decision criteria/alternative effects. Initially, decision-makers utilize Spherical Fuzzy Numbers (SFNs) to articulate their assessments in linguistic terms. These SFNs, reflecting the linguistic evaluations of each expert *H_t_*, form the core components of the Spherical Fuzzy Decision Matrix (SFDM) ${ℵ}_{H_{t}}$. For a comprehensive evaluation, it is necessary to establish *m* independent decision matrices ${ℵ}_{H_{1}},{ℵ}_{H_{2}},\ldots ,{ℵ}_{H_{m}}$, one for each expert on the panel. The SFDM corresponding to a specific expert *H_t_* is represented by Equation (8):

(8) $${ℵ}_{H_{t}}=\begin{array}{c} Z_{1}\\ Z_{2}\\ \vdots \\ Z_{p} \end{array}\begin{array}{cccccc} & T_{1} & T_{2} & \ldots & T_{p} & \\ ( & \begin{array}{c} \left(a_{11}^{\left(t\right)},b_{11}^{\left(t\right)},c_{11}^{\left(t\right)}\right)\\ \left(a_{21}^{\left(t\right)},b_{21}^{\left(t\right)},c_{21}^{\left(t\right)}\right)\\ \vdots \\ \left(a_{p1}^{\left(t\right)},b_{p1}^{\left(t\right)},c_{p1}^{\left(t\right)}\right) \end{array} & \begin{array}{c} \left(a_{12}^{\left(t\right)},b_{12}^{\left(t\right)},c_{12}^{\left(t\right)}\right)\\ \left(a_{22}^{\left(t\right)},b_{22}^{\left(t\right)},c_{22}^{\left(t\right)}\right)\\ \vdots \\ \left(a_{p2}^{\left(t\right)},b_{p2}^{\left(t\right)},c_{p2}^{\left(t\right)}\right) \end{array} & \begin{array}{c} \ldots \\ \ldots \\ \ddots \\ \ldots \end{array} & \begin{array}{c} \left(a_{2q}^{\left(t\right)},b_{2q}^{\left(t\right)},c_{2q}^{\left(t\right)}\right)\\ \left(a_{2q}^{\left(t\right)},b_{2q}^{\left(t\right)},c_{2q}^{\left(t\right)}\right)\\ \vdots \\ \left(a_{pq}^{\left(t\right)},b_{pq}^{\left(t\right)},c_{pq}^{\left(t\right)}\right) \end{array} & ) \end{array}$$

The SFN *F*(*t*) $F_{bg}^{\left(t\right)}=\left(a_{bg}^{\left(t\right)},b_{bg}^{\left(t\right)},c_{bg}^{\left(t\right)}\right)$ represents the judgment of expert *H_t_* depicting the caliber of alternative *Z_b_* under criterion *T_g_*. One vital thing that cannot be ignored is that if there are cost-oriented indicators in the index system, they need to be normalized.

Step 2: Construction of the aggregated SFDM.

In this step, the individual SFDMs are likely to be added together to provide the panel’s aggregate comments based on the experts’ weights. The aggregation is performed by applying the Spherical Weighted Arithmetic Mean (SWAM) operator, which accounts for the weights of the experts and ensures that the combined result preserves cumulative information from all inputs. The aggregated SFDM ℵ is constructed by accumulating the independent data, which can be represented using Equation (9). In the aggregated SFDM, each element represents the comprehensive evaluation of a specific alternative with respect to a specific criterion. For example, the *bg*-th entry $F_{bg}=\left(a_{bg}^{\left(t\right)},b_{bg}^{\left(t\right)},c_{bg}^{\left(t\right)}\right)$ corresponds to the *b*-th alternative under the *g*-th criterion, the value represents the aggregated evaluation of that alternative with respect to the criterion, incorporating the opinions of all experts weighted according to their respective importance. The detailed calculation method is given in Equation (10).

(9) $$ℵ=\begin{array}{c} Z_{1}\\ Z_{2}\\ \vdots \\ Z_{p} \end{array}\begin{array}{cccccc} & T_{1} & T_{2} & \ldots & T_{p} & \\ ( & \begin{array}{c} \left(a_{11},b_{11},c_{11}\right)\\ \left(a_{21},b_{21},c_{21}\right)\\ \vdots \\ \left(a_{p1},b_{p1},c_{p1}\right) \end{array} & \begin{array}{c} \left(a_{12},b_{12},c_{12}\right)\\ \left(a_{22},b_{22},c_{22}\right)\\ \vdots \\ \left(a_{p2},b_{p2},c_{p2}\right) \end{array} & \begin{array}{c} \ldots \\ \ldots \\ \ddots \\ \ldots \end{array} & \begin{array}{c} \left(a_{2q},b_{2q},c_{2q}\right)\\ \left(a_{2q},b_{2q},c_{2q}\right)\\ \vdots \\ \left(a_{pq},b_{pq},c_{pq}\right) \end{array} & ) \end{array}$$

(10) $$\begin{array}{l} F_{bg}=SWAM_{\kappa }\left(F_{bg}^{\left(1\right)},F_{bg}^{\left(2\right)},\ldots ,F_{bg}^{\left(m\right)}\right)=k_{1}F_{bg}^{\left(1\right)}⊕k_{2}F_{bg}^{\left(2\right)}⊕\cdots ⊕k_{m}F_{bg}^{\left(m\right)}\\ =(\sqrt{1-\prod_{t=1}^{m}{\left(1-a_{bg}^{{\left(t\right)}^{2}}\right)}^{k_{t}}},\sqrt{\prod_{t=1}^{m}{\left(1-a_{bg}^{{\left(t\right)}^{2}}\right)}^{k_{t}}-\prod_{t=1}^{m}{\left(1-b_{bg}^{{\left(t\right)}^{2}}-a_{bg}^{{\left(t\right)}^{2}}\right)}^{k_{t}}},\prod_{t=1}^{m}c_{bg}^{{\left(t\right)}^{2}k_{t}}). \end{array}$$

Step 3: Determination of criteria weights.

In this step, the SF-entropy method is utilized to evaluate the normalized criteria weights. The specifics of this evaluation are detailed in Section 3.2.

Step 4: Construction of the Weighted SFDM.

In this step, the relative importance of each criterion is incorporated into the aggregated evaluations, ensuring that the resulting matrix reflects both expert judgments and the significance of the criteria. Thus, the aggregated weighted SFDM $\overset{-}{ℵ}$ is constructed by multiplying the normalized weights of criteria with their respective aggregated SFDM terms. Each entry ${\bar{F}}_{bg}$ of $\overset{-}{ℵ}$ is obtained by Equation (11), where *ζ_g_* represents the weight of *g*th criterion.

(11) $${\bar{F}}_{bg}=\zeta_{g}F_{bg}=\left(\sqrt{\left(1-{\left(1-a_{bg}^{2}\right)}^{\zeta g}\right)},\right.\left.\sqrt{\left({\left(1-a_{bg}^{2}\right)}^{\zeta g}-{\left(1-a_{bg}^{2}-b_{bg}^{2}\right)}^{\zeta g}\right)},\;\;\;{\left(c_{bg}\right)}^{\zeta g}\right)$$

Step 5: Construction of concordance and discordance sets.

Drawing on the foundational elements of ELECTRE methodologies, the presented approaches evaluate the pairwise relationships among alternatives, focusing on their comparative merits and deficiencies. To delineate the dominance or disadvantage of one alternative over another based on decision criteria, the established concepts of spherical fuzzy concordance sets (SFCSs) and spherical fuzzy discordance sets (SFDSs) are employed. The performance of alternatives is compared pairwise using scores and degrees of accuracy derived from spherical fuzzy inputs within the consolidated weighted Spherical Fuzzy Decision Matrix (SFDM) models.

To highlight differences in superiority, SFCSs are categorized into two distinct types: one known as the spherical fuzzy set of strong concordance (SFSCs) and the other termed the spherical fuzzy set of weak concordance (SFWCS). The principal criteria for forming SFCSs to reveal primacy between two solutions involve the following:

SFSCS refers to the collection of criteria where one alternative exhibits a distinct and significant superiority over another. It captures the circumstances in which the dominance of one solution is evident and undeniable, thereby representing the strictest form of pairwise superiority. In this context, the SFSCS highlights only those criteria where the superiority of alternative *Z_b_* over alternative *Z_r_* is strong, consistent, and substantial. The mathematical condition for establishing SFSCS is presented in Equation (12).

(12) $${ℑ}_{br}^{s}=\{g:S({\overset{-}{F}}_{bg})>S({\overset{-}{F}}_{rg}),A({\overset{-}{F}}_{bg})\geq A({\overset{-}{F}}_{rg})\},b,r=1,2,\ldots ,p,b\neq r.$$

SFWCS refers to the collection of criteria where one alternative demonstrates only a slight or marginal advantage over another. It captures the cases in which superiority exists but remains limited in strength, representing a weaker form of pairwise prominence. In this context, the SFWCS highlights situations where alternative *Z_b_* has a modest or partial edge over alternative *Z_r_*, rather than a strong dominance. The condition for establishing SFWCS is expressed in Equation (13).

(13) $${ℑ}_{br}^{w}=\{g:S({\overset{-}{F}}_{bg})\geq S({\overset{-}{F}}_{rg}),A({\overset{-}{F}}_{bg})<A({\overset{-}{F}}_{rg})\},b,r=1,2,\ldots ,p,b\neq r.$$

Additionally, it is essential to assess the alternatives from an opposite perspective, referred to as discordance. In this regard, SFDSs act as the inverse counterparts of the SFCSs. These sets are specifically designed to capture the weaknesses or disadvantages of one alternative when compared to another. The severity of such shortcomings depends on the extent of performance disparities, leading to a further classification into Spherical Fuzzy Strong Discordance Sets (SFSDSs) and Spherical Fuzzy Weak Discordance Sets (SFWDSs). The fundamental conditions for constructing SFDSs are defined as follows:

SFSDS refers to the collection of criteria where one alternative demonstrates a clear and substantial inferiority compared to another. It captures the circumstances in which the weaknesses of one solution are evident and undeniable, thus representing the strictest form of subordination in pairwise comparison. In this context, the SFSDS highlights only those criteria where alternative *Z_b_* is strongly and consistently inferior to alternative *Z_r_*. The formal condition for establishing SFSDS is given in Equation (14).

(14) $${ℜ}_{br}^{w}=\{g:S({\overset{-}{F}}_{bg})\leq S({\overset{-}{F}}_{rg}),A({\overset{-}{F}}_{bg})<A({\overset{-}{F}}_{rg})\},b,r=1,2,\ldots ,p,b\neq r.$$

SFWDS refers to the collection of criteria where one alternative exhibits only slight or marginal shortcomings compared to its competitor. It reflects a weaker form of inefficacy, emphasizing situations in which the disadvantages exist but remain limited in impact. The SFWDS therefore represents the cases where alternative *Z_b_* shows modest or partial inferiority relative to alternative *Z_r_*. The formal condition for constructing SFWDS is defined in Equation (15).

(15) $${ℜ}_{br}^{w}=\{g:S({\overset{-}{F}}_{bg})<S({\overset{-}{F}}_{rg}),A({\overset{-}{F}}_{bg})\geq A({\overset{-}{F}}_{rg})\},b,r=1,2,\ldots ,p,b\neq r.$$

Step 6: Formation of the spherical fuzzy discordance matrix.

In the last step, SFCSs and SFSDSs were constructed. Based on SFSDSs, the discordance index *η_br_*, is introduced to reveal the extent of inferiority of alternative *Z_b_* relative to alternative *Z_r_*. Specifically, *η_br_* measures the degree to which *Z_b_* is less favorable compared to *Z_r_* across multiple criteria, it is calculated based on the weighted distances between the compared alternatives concerning each criterion. A higher value indicates that *Z_b_* is significantly weaker in one or more aspects, highlighting its competitive shortcomings. The discordance index can be calculated based on Equation (16). The weights of the SFWDS and SFSDS are assigned to $k_{ℜ}^{s}$ and $k_{ℜ}^{w}$, respectively.

(16) $$\eta_{br}=\frac{\max\{\max_{g\in {ℜ}_{br}^{s}}\left(k_{ℜ}^{s}\times d\left({\bar{F}}_{bg},{\bar{F}}_{rg}\right)\right),\max_{g\in {ℜ}_{br}^{w}}\left(k_{ℜ}^{w}\times d\left({\bar{F}}_{bg},{\bar{F}}_{rg}\right)\right)\}}{\max_{g}d\left({\bar{F}}_{bg},{\bar{F}}_{rg}\right)}.$$

where $d\left({\bar{F}}_{bg},{\bar{F}}_{rg}\right)$ represents the Euclidean distance between two SFNs ${\bar{F}}_{bg}$ and ${\bar{F}}_{rg}$, which is derived from the aggregated weighted SFDM obtained in Step 4. This distance can be calculated using Equation (17).

(17) $$d\left({\bar{F}}_{bg},{\bar{F}}_{rg}\right)=\sqrt{\left({\left({\bar{a}}_{bg}-{\bar{a}}_{\mathrm{rg}}\right)}^{2}+{\left({\bar{b}}_{bg}-{\bar{b}}_{rg}\right)}^{2}+{\left({\bar{c}}_{bg}-{\bar{c}}_{\mathrm{rg}}\right)}^{2}\right)}.$$

By systematically evaluating *η_br_* for all pairs of alternatives, the spherical fuzzy discordance matrix (SFDM) can then be constructed as shown in Equation (18). This matrix is a square array where each element corresponds to the discordance index between a pair of alternatives, summarizing the relative inferiority of all alternatives in a comprehensive form. For example, *η*_12_ represents the discordance index of alternative *Z*_1_ relative to *Z*_2_, indicating the degree to which *Z*_1_ is relatively disadvantaged compared to *Z*_2_.

(18) $$\eta =\begin{array}{c} Z_{1}\\ Z_{2}\\ \vdots \\ Z_{p} \end{array}\begin{array}{cccccc} & Z_{1} & Z_{2} & \ldots & Z_{p} & \\ ( & \begin{array}{c} -\\ \eta_{21}\\ \vdots \\ \eta_{p1} \end{array} & \begin{array}{c} \eta_{12}\\ -\\ \vdots \\ \eta_{p2} \end{array} & \begin{array}{c} \ldots \\ \ldots \\ \ddots \\ \ldots \end{array} & \begin{array}{c} \eta_{1p}\\ \eta_{2p}\\ \vdots \\ - \end{array} & ) \end{array}$$

Step 7: Formation of the spherical fuzzy concordance matrix.

In this step, the concordance index is calculated by utilizing the SFCSs obtained in step 5. While discordance indices measure the relative inferiority of alternatives, concordance indices quantify their relative superiority across multiple criteria. Specifically, for a pair of alternatives (*Z_b_*, *Z_r_*), the concordance index measures the degree to which *Z_b_* is more favorable compared to *Z_r_*. It is calculated based on the weighted sum of the normalized weights of the criteria found within the respective categories of the concordance sets, as shown in Equation (19). The weights of the SFWCS and SFSCS are assigned to $k_{ℑ}^{w}$ and $k_{ℑ}^{c}$, respectively.

(19) $$\vartheta_{br}=k_{ℑ}^{s}\sum_{g\in {ℑ}_{br}^{s}}\zeta_{g}+k_{ℑ}^{w}\sum_{g\in {ℑ}_{br}^{w}}\zeta_{g}.$$

By systematically evaluating all concordance indices for every pair of alternatives, the spherical fuzzy concordance matrix (SFCM) ϑ can be constructed as shown in Equation (20). This matrix is a square array where each element corresponds to the concordance index between a pair of alternatives, summarizing the relative superiority of all alternatives in a comprehensive form. For example, $\vartheta_{12}$ represents the concordance index of alternative *Z*_1_ relative to *Z*_2_, indicating the degree to which *Z*_1_ is more superior compared to *Z*_2_.

(20) $$\vartheta =\begin{array}{c} Z_{1}\\ Z_{2}\\ \vdots \\ Z_{p} \end{array}\begin{array}{cccccc} & Z_{1} & Z_{2} & \ldots & Z_{p} & \\ ( & \begin{array}{c} -\\ \vartheta_{21}\\ \vdots \\ \vartheta_{p1} \end{array} & \begin{array}{c} \vartheta_{12}\\ -\\ \vdots \\ \vartheta_{p2} \end{array} & \begin{array}{c} \ldots \\ \ldots \\ \ddots \\ \ldots \end{array} & \begin{array}{c} \vartheta_{1p}\\ \vartheta_{2p}\\ \vdots \\ - \end{array} & ) \end{array}$$

Step 8: Construction of spherical fuzzy effective concordance matrix.

In this phase, the concordance indices obtained in Step 7 are evaluated against a predefined threshold to determine whether one alternative can be considered clearly superior to the alternative it is compared against. Unlike Step 7, which calculates continuous concordance indices reflecting the degree of superiority, the main purpose of this step is to establish a clear decision criterion. This involves defining a benchmark or threshold value, known as the concordance level *ρ*, to assess whether the concordance index is sufficiently high. Essentially, the concordance level represents the minimum concordance required for one alternative to be recognized as more favorable than its competitor. The concordance level can be obtained using Equation (21).

(21) $$\rho =\frac{1}{p(p-1)}\sum_{b=1,b\neq r}^{p}\sum_{r=1,r\neq b}^{p}\vartheta_{br}.$$

Next, each pair of alternatives is assigned a binary value to indicate effective superiority. Specifically, if the concordance index for a pair of alternatives (*Z_b_*, *Z_r_*) meets or exceeds the threshold *ρ*, it is assigned a value of 1, indicating that *Z_b_* can be considered superior to *Z_r_*. Conversely, if the concordance index falls below the threshold, it is assigned a value of 0, indicating non-superiority. This binary evaluation can be formally expressed as

(22) $${\tilde{\vartheta }}_{br}=\left\{\begin{array}{ll} 1, & \;\;\mathrm{if}\;\vartheta_{br}\geq \rho \\ 0, & \;\;\mathrm{if}\;\vartheta_{br}<\rho \end{array}\right.$$

The effective spherical fuzzy concordance matrix $\tilde{\vartheta }$ is assembled by dominant or effective concordance, which can be obtained by Equation (23).

(23) $$\tilde{\vartheta }=\begin{array}{c} Z_{1}\\ Z_{2}\\ \vdots \\ Z_{p} \end{array}\begin{array}{cccccc} & Z_{1} & Z_{2} & \ldots & Z_{p} & \\ ( & \begin{array}{c} -\\ {\tilde{\vartheta }}_{21}\\ \vdots \\ {\tilde{\vartheta }}_{p1} \end{array} & \begin{array}{c} {\tilde{\vartheta }}_{12}\\ -\\ \vdots \\ {\tilde{\vartheta }}_{p2} \end{array} & \begin{array}{c} \ldots \\ \ldots \\ \ddots \\ \ldots \end{array} & \begin{array}{c} {\tilde{\vartheta }}_{1p}\\ {\tilde{\vartheta }}_{2p}\\ \vdots \\ - \end{array} & ) \end{array}$$

Step 9: Construction of spherical fuzzy effective discordance matrix.

Similar to the evaluation of concordance indices, this step assesses the discordance indices against a predefined discordance level l. The discordance level represents the minimal value that confirms one alternative outranks another based on discordance information. In simpler terms, pairs with discordance indices below this threshold contribute to establishing outranking relations. The discordance level can be obtained by Equation (24).

(24) $$l=\frac{1}{p(p-1)}\sum_{b=1,b\neq r}^{p}\sum_{r=1,r\neq b}^{p}\eta_{br}$$

The following show the method to obtain the effective discordance indices.

Each pair of alternatives is assigned a binary value based on the discordance threshold. If the discordance index is below l, it is assigned a value of 1; otherwise, it is assigned a value of 0. This can be formally expressed as

(25) $${\tilde{\eta }}_{br}=\left\{\begin{array}{ll} 1, & \;\;\mathrm{if}\;\eta_{br}\leq l;\\ 0, & \;\;\mathrm{if}\;\eta_{br}>l; \end{array}\right.$$

The spherical fuzzy effective discordance matrix $\tilde{\eta }$ can be obtained by Equation (26).

(26) $$\tilde{\eta }=\begin{array}{c} Z_{1}\\ Z_{2}\\ \vdots \\ Z_{p} \end{array}\begin{array}{cccccc} & Z_{1} & Z_{2} & \ldots & Z_{p} & \\ ( & \begin{array}{c} -\\ {\tilde{\eta }}_{21}\\ \vdots \\ {\tilde{\eta }}_{p1} \end{array} & \begin{array}{c} {\tilde{\eta }}_{12}\\ -\\ \vdots \\ {\tilde{\eta }}_{p2} \end{array} & \begin{array}{c} \ldots \\ \ldots \\ \ddots \\ \ldots \end{array} & \begin{array}{c} {\tilde{\eta }}_{1p}\\ {\tilde{\eta }}_{2p}\\ \vdots \\ - \end{array} & ) \end{array}$$

Step 10: Construction of spherical fuzzy aggregated dominance matrix.

By combining the effective concordance and effective discordance matrices, the spherical fuzzy aggregated dominance matrix (SFADM) *τ* is obtained to represent the overall superiority of alternatives. Specifically, an alternative is considered superior to another only if it is effective in both concordance and discordance. For example, if both the effective concordance and discordance values for a pair are 1, the first alternative is regarded as superior to the second. The SFADM can be formally computed using Equation (27) and represented in matrix form as shown in Equation (28).

(27) $$\tau_{br}={\tilde{\eta }}_{br}\cdot {\tilde{\vartheta }}_{br}$$

(28) $$\tau =\begin{array}{c} Z_{1}\\ Z_{2}\\ \vdots \\ Z_{p} \end{array}\begin{array}{cccccc} & Z_{1} & Z_{2} & \ldots & Z_{p} & \\ ( & \begin{array}{c} -\\ \tau_{21}\\ \vdots \\ \tau_{p1} \end{array} & \begin{array}{c} \tau_{12}\\ -\\ \vdots \\ \tau_{p2} \end{array} & \begin{array}{c} \ldots \\ \ldots \\ \ddots \\ \ldots \end{array} & \begin{array}{c} \tau_{1p}\\ \tau_{2p}\\ \vdots \\ - \end{array} & ) \end{array}$$

Step 11: Construction of the Outranking Graph.

The SF-ELECTRE method assesses alternatives by analyzing the outranking relations derived from the Spherical Fuzzy Aggregated Dominance Matrix (SFADM). To provide a clear visualization of these relations, an outranking graph is constructed in which each vertex represents an alternative. Directed arcs between vertices indicate the existence and direction of outranking relationships. The construction of the outranking graph follows the principles outlined below:

If *τ_rb_* = 0 and *τ_br_* = 1, signifying that alternative *Z_b_* has enough capacity to outrun *Z_r_*, then an arc can be drawn from *Z_b_* pointing to *Z_r_*.

If *τ_rb_* = 1 and *τ_br_* = 1, signifying that a mutual outranking relationship between alternatives *Z_b_* and *Z_r_*, then a double-sided arrow arc can be drawn between them.

If *τ_rb_* = 0 and *τ_br_* = 0, signifying that there remains no relationship between alternatives, then no arc needs to be drawn among alternatives.

### 3.4. The Framework of the Proposed Method

**Figure 2:**
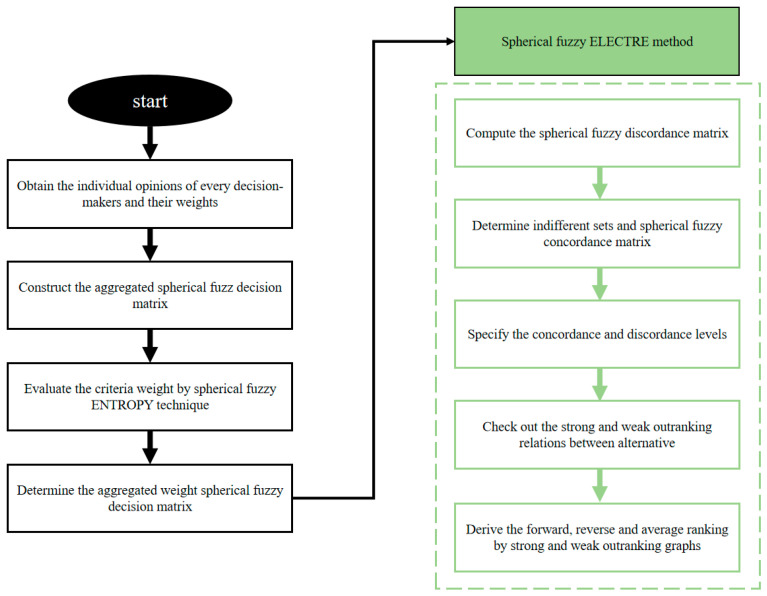
The flowchart diagram.

The integrated flowchart of the two scenarios is shown in Figure 2. Three experts from the area of intelligent automobile cockpits are invited to participate in the evaluation and provide their decision information. The decision information is provided in the form of SFNs from the experts. The criterion weights are further computed using the spherical fuzzy entropy method. The aggregated operation of SFSs is introduced to merge the criterion and decision matrix. In addition, the spherical fuzzy ELECTRE method is proposed to determine the optimal alternatives.

## 4. Case Study

### 4.1. Background

To assess the reasonableness of the model, engineering examples are also needed to assist in validation [53]. In fact, in the process of car intelligence and electrification, car companies have launched intelligent cockpits equipped with car systems through independent research and development, partner support, system integration, and other means one after another [54].

Three kinds of intelligent automobile bionic cockpits, as empirical applications, are adopted to verify the application of the proposed solution method. The first alternative is Oshan Z6 Smart Happy Cockpit, which relies on the Onstyle 5.0 car computer system to solve the needs of users in diversified travel scenarios. The system has the characteristics of high sensitivity, accurate feedback, super smoothness, and fast iteration in terms of arithmetic power. In contrast, the AITO M5 is positioned as a technologically advanced alternative, whose conceptual foundation is centered on the Har-monyOS system. The intelligent cockpit of the third alternative is XPeng intelligent cockpit. It is characterized by a collection of technological attributes of the configuration. Through intelligent car software and hardware systems, technology, and entertainment configuration, etc., present the intelligent third space under the entertainment scene, sleep scene, outdoor scene, life scene, and DIY scene.

These three cockpit types were deliberately selected because they represent mainstream technological directions in the current automotive industry. Alternative 1 reflects the computing power-driven cockpit, characterized by high responsiveness and efficient algorithmic performance. Alternative 2 embodies the ecosystem-driven cockpit, in which HarmonyOS-based interconnection provides a representative example of cross-device integration that has already been adopted by multiple automobile manufacturers in China. Alternative 3 highlights the scenario-driven cockpit, integrating software-hardware coordination to support diversified user experiences such as entertainment, sleep, and outdoor functions, which reflects the growing trend of positioning the cockpit as a “third living space.” Collectively, these three cases cover the major development paradigms of intelligent cockpits. This ensures that the evaluation results are broadly applicable, providing insights that are useful for both engineering design and market analysis.

All three categories of intelligent cockpits are centered on the needs of users’ diversified travel scenarios, providing intelligent and technological solutions. In this paper, based on a combination of factors, these three intelligent automobile cockpits are introduced to illustrate the proposed methods, and the cockpits are named Alternative 1, Alternative 2, and Alternative 3.

### 4.2. Weighting of Criteria via Fuzzy Entropy Method

**Figure 3:**
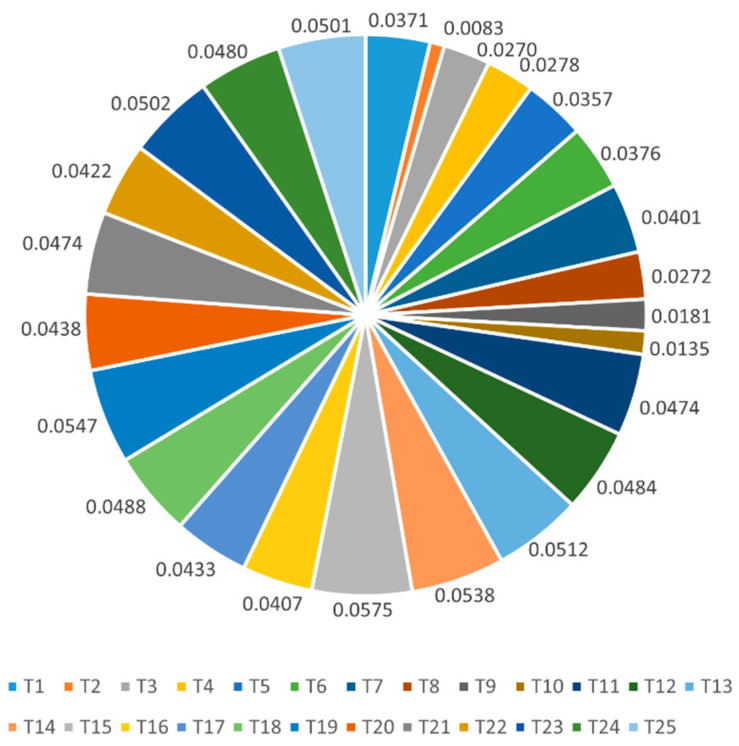
Weights of each criterion.

For criteria considered in the decision process, the weight of each criterion represents its importance for decision-making. To reduce the subjectivity of experts, the entropy combined with SFSs is introduced to obtain the criterion weights. Based on the case background in Section 4.1, three experts are engaged to provide decision information about the three alternatives over each criterion, and the decision matrix is expressed in the form of SFNs. The criterion weights are computed using Equations (4)–(7), and the results are shown in Figure 3.

It can be found that T_15_ is the most important criterion, and criterion T_2_ shows the lowest importance in the selection of intelligent automobile cockpits. In addition, the weights of T_19_, T_14_, T_13_, T_23_, and T_25_ are above 0.05, which represents the important criteria. While criteria T_10_, T_9_, T_3_, T_8_, and T_4_ can be viewed as unimportant. It shows that human–computer interaction, early warning systems, and multimedia entertainment systems have received a lot of attention from drivers and passengers. Some criteria in voice, light, and heat are not the main considerations for drivers and passengers.

### 4.3. Obtaining the Optimal Alternative of Intelligent Automobile Cockpit

Step-by-step solutions to the depicted problem are estimated in the following steps:

Step 1: The efficiency of the different intelligent cockpits is depicted by defining the representable linguistic terms in SFNs, as shown in Table 1. Table A1 represents the linguistic preferences for experts. The linguistic scale consisting of “Very High”, “High”, “Average”, “Low”, and “Very Low” was determined with reference to previous fuzzy MCDM studies [55,56]. This structure ensures sufficient discrimination among alternatives while keeping the evaluation process understandable and computationally efficient for experts. The SFDMs, ${ℵ}_{H_{1}}$,${ℵ}_{H_{2}}$ and ${ℵ}_{H_{3}}$ of the experts $H_{1}$, $H_{2}$ and $H_{3}$ are represented by Table A2, Table A3 and Table A4, respectively. Due to the existence of cost-type indexes in the established index system, the normalized individual SFDMs $ℵ\prime_{H_{1}}$, $ℵ\prime_{H_{2}}$, $ℵ\prime_{H_{3}}$ are shown in Table A5, Table A6 and Table A7.

Step 2: Compute the aggregated SFDMs by operating on the entries of the individual SFDMs using Equation (9), as shown in Table A8.

Step 3: Utilize SF-entropy method to settle the criterion weights, the results are shown in Figure 3.

Step 4: Multiply the normalized criterion weights with the specified aggregated SFDM columns based on Equation (11). The results are collapsed into a weighted SFDM as shown in Table A9.

Step 5: Alternatives are evaluated under each criterion based on the accuracy and score degrees of their respective entries within the aggregated weighted SFDM. Table A10 and Table A11 shows the details of the score and score accuracy degrees of the SFNs in the aggregated weighted SFDM.

For each pair of compared alternatives, weak and strong concordance sets are determined based on the criteria specified in Equations (12) and (13). Table A12 and Table 2 show the entries for the strong concordance sets and the weak concordance sets, respectively. Furthermore, strong discordance sets are compiled in accordance with Equation (14) and are presented in Table A13. The weak discordance sets are recorded in Table 3 using Equation (15).

Step 6: To evaluate discordance indices, the distance measures between alternatives with respect to all criteria are computed using Equation (16). Table A14 presents the Euclidean distance among the alternative with respect to all considered criteria. For the computation of discordance indices, the respective weights of SFSDSs $k_{ℑ}^{s}$ and SFWDSs $k_{ℑ}^{s}$ are taken as 0.75 and 1.0 by the decision-makers. Finally, the discordance indices, computed by Equation (18), are assembled to form the SFDM as follows:

(29) $$\eta =\begin{array}{c} Z_{1}\\ Z_{2}\\ Z_{3} \end{array}\begin{array}{ccccc} & Z_{1} & Z_{2} & Z_{3} & \\ ( & \begin{array}{c} -\\ 0.3355\\ 0.4791 \end{array} & \begin{array}{c} 1.0\\ -\\ 0.4037 \end{array} & \begin{array}{c} 1.0\\ 0.2663\\ - \end{array} & ) \end{array}$$

Step 7: The SFCM is built by arranging the concordance indices utilizing Equation (30).

(30) $$\vartheta =\begin{array}{c} Z_{1}\\ Z_{2}\\ Z_{3} \end{array}\begin{array}{ccccc} & Z_{1} & Z_{2} & Z_{3} & \\ ( & \begin{array}{c} -\\ 0.6092\\ 0.65 \end{array} & \begin{array}{c} 0.1406\\ -\\ 0.4606 \end{array} & \begin{array}{c} 0.1958\\ 0.3477\\ - \end{array} & ) \end{array}$$

Step 8: The concordance level and SFCDM can be obtained by Equation (31) and Equation (32), respectively.

(31) $$\rho =\frac{1}{3\times 2}(0.1406+0.1958+0.6092+0.3477+0.65+0.4606)=0.4006$$

(32) $$\tilde{\vartheta }=\begin{array}{c} Z_{1}\\ Z_{2}\\ Z_{3} \end{array}\begin{array}{ccccc} & Z_{1} & Z_{2} & Z_{3} & \\ ( & \begin{array}{c} -\\ 1\\ 1 \end{array} & \begin{array}{c} 0\\ -\\ 1 \end{array} & \begin{array}{c} 0\\ 0\\ - \end{array} & ) \end{array}$$

Step 9: The discordance level and SFDDM can be obtained by Equations (33) and (34).

(33) $$l=\frac{1}{3\times 2}(1.0+1.0+0.3355+0.2663+0.4791+0.4037)=0.5808$$

(34) $$\tilde{\eta }=\begin{array}{c} Z_{1}\\ Z_{2}\\ Z_{3} \end{array}\begin{array}{ccccc} & Z_{1} & Z_{2} & Z_{3} & \\ ( & \begin{array}{c} -\\ 1\\ 1 \end{array} & \begin{array}{c} 0\\ -\\ 1 \end{array} & \begin{array}{c} 0\\ 0\\ - \end{array} & ) \end{array}$$

Step 10: Obtain the SFADM by Equation (35).

(35) $$\tau =\begin{array}{c} Z_{1}\\ Z_{2}\\ Z_{3} \end{array}\begin{array}{ccccc} & Z_{1} & Z_{2} & Z_{3} & \\ ( & \begin{array}{c} -\\ 1\\ 1 \end{array} & \begin{array}{c} 0\\ -\\ 1 \end{array} & \begin{array}{c} 0\\ 0\\ - \end{array} & ) \end{array}$$

**Figure 4:**
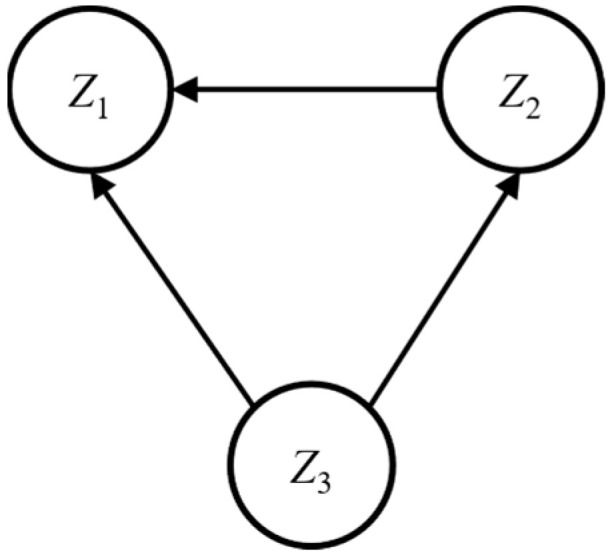
Outranking graph of the three intelligent automobile cockpits.

Step 11: A prioritization graph is drawn utilizing the SFADM to show the pairwise relationships among the alternatives, which is represented by Figure 4, where *Z*_1_ represents Alternative 1, *Z*_2_ represents Alternative 2, and *Z*_3_ represents Alternative 3. The optimal alternative can be obtained by extracting information from the ranking graph, where the extracted information is shown in Table 4. It can be found that *Z*_3_ is selected as the optimal alternative, which means XPeng intelligent cockpit is the best according to SF-ELECTRE method.

## 5. Validation and Sensitivity Analysis

### 5.1. Validation

To illustrate the validation and features of the above-mentioned methods, a comprehensive comparative experiment was conducted using several well-established MCDM algorithms. In particular, the TODIM method was applied under different behavioral coefficients (θ) to simulate diverse risk attitudes of decision-makers, including extremely risk-seeking (θ=0.01), moderately risk-seeking (θ=0.1), risk-neutral (θ=1), typically loss-averse (θ=5), and highly loss-averse (θ=10) scenarios. These settings comprehensively capture the influence of psychological behavior and decision attitude on ranking outcomes. In addition, the VIKOR method was examined under different risk decision factors, including the regret-averse strategy (ς=0.25), the balanced strategy (ς=0.5), and the utility-maximizing strategy (ς=0.75), to investigate its sensitivity to varying degrees of risk preference. Furthermore, three additional classical approaches: TOPSIS, CoCoSo, and SAW were employed to provide a multi-perspective validation of the proposed method’s stability and discriminative capacity. The same weights are given to the above methods to make sure the results will not be affected by different criterion weights.

The results are shown in Table 5, the numerical values represent the ranking order of alternatives (1 = best, 3 = worst). It can be found that the results of TOPSIS are the same as the SFS-ELECTRE method. The optimal alternative is Alternative 3 and the worst alternative is Alternative 1, confirming the rational consistency and reliability of the proposed approach. In contrast, the TODIM method consistently produces the ranking order Alternative 2 > Alternative 1 > Alternative 3 under all behavioral coefficient settings, indicating Alternative 2 may appear favorable due to its balanced performance across criteria, leading decision-makers to perceive relatively high net gains during comparisons. For the VIKOR method, different risk decision factors lead to slight variations in the ranking outcomes, reflecting its ability to capture decision-makers’ risk preferences. Specifically, under the regret-averse strategy (ς=0.25) and a balanced strategy (ς=0.5), Alternative 1 is ranked as the best, followed by Alternative 3 and Alternative 2. In contrast, under the utility-maximizing strategy (ς=0.75), Alternative 1 remains the best, but Alternative 2 rises to the second position, and Alternative 3 becomes the least preferred. These differences indicate that VIKOR’s results are influenced by the decision-makers’ risk attitudes. Furthermore, both CoCoSo and SAW yield identical ranking results, with Alternative 1 ranked first, Alternative 2 s, and Alternative 3 last, which is due to CoCoSo being constructed based on the principles of both SAW and TOPSIS.

It is worth noting that although TOPSIS and the proposed SFS-ELECTRE method yield consistent rankings, their decision mechanisms differ fundamentally. TOPSIS is compensatory, allowing poor performance in one criterion to be offset by strengths in others, whereas SFS-ELECTRE employs non-compensatory outranking logic, preventing critical weaknesses from being fully offset. This is especially relevant for intelligent cockpit evaluation, where safety, comfort, and entertainment are not interchangeable. Combined with spherical fuzzy sets, the method better captures uncertainty and enhances interpretability. Thus, even with similar rankings, the SFS-ELECTRE framework offers a more realistic decision rationale and better reflects the non-compensatory nature of such evaluations.

### 5.2. Sensitivity Analysis

To verify the stability of the proposed method in the decision-making process, the sensitivity analysis of criterion weights is carried out. For each criterion, the weight value is cut by 10%, while the weights of the rest criteria remain unchanged. With the unchanged decision processes of the ELECTRE method, the corresponding decision results of each processed criterion group can be obtained, as shown in Table 6. The results show that Alternative 3 is the optimal alternative and Alternative 1 is the worst alternative in all the criterion groups. In addition, the ranking order of the three alternatives remains the same with different processed criteria. It can be found that the fluctuated criteria weights have little influence on the decision results. The decision approach proposed in this study exhibits high stability.

## 6. Conclusions

Intelligent automobile bionic cockpits have great potential in serving drivers and ensuring safety. In recent years, the personalization design of such cockpits has attracted considerable attention. However, accurately describing passenger personalization needs and systematically evaluating intelligent cockpits remain challenging. To address these issues, this study analyzes passengers’ needs in terms of security, comfort, and spiritual entertainment, and proposes a multiple-criterion decision-making framework that integrates spherical fuzzy sets, entropy weighting, and the ELECTRE method. A case study of three representative cockpits demonstrates that the approach can effectively distinguish among alternatives, identifying Alternative 3 as the optimal choice and Alternative 1 as the least favorable. Finally, a comparative analysis including TODIM, VIKOR, and the classical methods TOPSIS, CoCoSo, and SAW shows that TODIM and VIKOR reflect behavioral or risk preferences and the others are compensatory, SFS-ELECTRE provides a stable, rational ranking, integrates spherical fuzzy sets to handle uncertainty, and employs a non-compensatory logic, making it a robust and interpretable tool for evaluating alternatives with non-substitutable criteria.

However, the three cases do not cover all cockpit types and configurations, and the method developed for intelligent automobile bionic cockpits may have limited applicability to conventional cars, trucks, or buses. Future studies could expand the sample diversity and further explore the method’s applicability across different vehicle types. In addition, the proposed method could be integrated into computer-supported collaborative decision-making systems, enabling designers, engineers, and decision-makers to collaboratively evaluate and select intelligent automobile bionic cockpits with higher efficiency and real-time feedback.

## Figures and Tables

**Table 1:** The linguistic terms.

Linguistic Term	Abbreviations	Fuzzy Rating
Very high	VL	(0.86, 0.23, 0.20)
High	H	(0.75, 0.31, 0.37)
Average	A	(0.59, 0.50, 0.55)
Low	L	(0.37, 0.31, 0.75)
Very low	VL	(0.20, 0.12, 0.94)

**Table 2:** Weak concordance sets.

	*Z*_1_	*Z*_2_	*Z*_3_
*Z*_1_	{}	{1, 2, 3, 7, 9, 12, 13, 15, 16, 17, 18, 19, 20, 22, 24}	{1, 3, 7, 9, 12, 15, 16, 17, 18, 19, 20, 22, 23, 24, 25}
*Z*_2_	{5, 6, 10, 14}	{}	{5, 6, 7, 10, 15, 16, 17, 20, 23, 24, 25}
*Z*_3_	{4, 5, 8, 13, 14}	{1, 2, 3, 4, 8, 9, 12, 13, 14, 18}	{}

**Table 3:** Weak discordance sets.

	*Z*_1_	*Z*_2_	*Z*_3_
*Z*_1_	{}	{5, 6, 10, 14}	{4, 5, 8, 13, 14}
*Z*_2_	{1, 2, 3, 7, 9, 12, 13, 15, 16, 17, 18, 19, 20, 22, 24}	{}	{1, 2, 3, 4, 8, 9, 12, 13, 14, 18}
*Z*_3_	{1, 3, 7, 9, 12, 15, 16, 17, 18, 19, 20, 22, 23, 24, 25}	{5, 6, 7, 10, 15, 16, 17, 20, 23, 24, 25}	{}

**Table 4:** Analysis of outranking graph.

Alternatives	Submissive Alternatives	Incomparable Alternatives
*Z*_1_	-	-
*Z*_2_	*Z*_1_	*Z*_3_
*Z*_3_	*Z*_1_, *Z*_2_	-

**Table 5:** The results of validation.

Linguistic Term	Alternative 1 (Rank)	Alternative 2 (Rank)	Alternative 3 (Rank)
SFS-ELECTRE	3	2	1
TODIM (θ=0.01)	2	1	3
TODIM (θ=0.1)	2	1	3
TODIM (θ=1)	2	1	3
TODIM (θ=5)	2	1	3
TODIM (θ=10)	2	1	3
VIKOR (ς=0.25)	1	3	2
VIKOR (ς=0.5)	1	3	2
VIKOR (ς=0.75)	1	2	3
TOPSIS	3	2	1
CoCoSo	1	2	3
SAW	1	2	3

**Table 6:** Results of sensitivity analysis.

Criterion	Alternative 1	Alternative 2	Alternative 3	Criterion	Alternative 1	Alternative 2	Alternative 3
T_1_	3	2	1	T_14_	3	2	1
T_2_	3	2	1	T_15_	3	2	1
T_3_	3	2	1	T_16_	3	2	1
T_4_	3	2	1	T_17_	3	2	1
T_5_	3	2	1	T_18_	3	2	1
T_6_	3	2	1	T_19_	3	2	1
T_7_	3	2	1	T_20_	3	2	1
T_8_	3	2	1	T_21_	3	2	1
T_9_	3	2	1	T_22_	3	2	1
T_10_	3	2	1	T_23_	3	2	1
T_11_	3	2	1	T_24_	3	2	1
T_12_	3	2	1	T_25_	3	2	1
T_13_	3	2	1				

## Data Availability

Data are contained within the article.

## Appendix A

**Table A1:** Linguistic preferences for experts.

Decision-Makers	Alter.	*T*_1_	*T*_2_	*T*_3_	*T*_4_	*T*_5_	*T*_6_	*T*_7_	*T*_8_	*T*_9_	*T*_10_	*T*_11_	*T*_12_	*T*_13_
Z_1_	Z_1_	H	A	A	H	H	H	A	A	A	L	L	H	VH
	Z_2_	L	A	L	H	H	H	VH	A	L	A	L	H	VH
	Z_3_	A	H	A	A	H	A	H	A	A	A	L	H	A
Z_2_	Z_1_	A	A	L	A	VH	H	A	H	A	A	L	H	A
	Z_2_	L	A	L	A	H	L	H	H	A	A	L	H	H
	Z_3_	L	A	A	A	A	L	H	H	A	A	L	VH	VH
Z_3_	Z_1_	A	A	H	H	H	A	H	L	A	A	L	H	H
	Z_2_	VL	L	L	H	A	H	H	L	L	A	L	VH	VH
	Z_3_	L	A	L	H	H	H	VH	A	L	L	L	A	L
**Decision-Makers**	**Alter.**	***T*_14_**	***T*_15_**	***T*_16_**	***T*_17_**	***T*_18_**	***T*_19_**	***T*_20_**	***T*_20_**	***T*_22_**	***T*_23_**	***T*_24_**	***T*_25_**	
Z_1_	Z_1_	VH	L	A	A	L	L	H	H	A	H	L	H	
	Z_2_	H	VH	H	H	VH	VH	H	H	H	H	VH	VH	
	Z_3_	A	H	VH	VH	H	VH	A	H	H	H	H	H	
Z_2_	Z_1_	VH	H	H	A	L	L	H	H	H	H	A	H	
	Z_2_	VH	H	H	H	VH	VH	VH	H	H	H	H	VH	
	Z_3_	VH	H	H	VH	H	VH	H	H	H	VH	H	H	
Z_3_	Z_1_	H	VL	L	L	A	A	A	H	H	H	VH	H	
	Z_2_	H	L	A	H	H	H	A	H	H	H	H	H	
	Z_3_	L	VH	H	H	H	H	VH	H	H	H	VH	VH	

**Table A2:** The FSMD of expert *H*_1_.

*H*_1_	*T*_1_	*T*_2_	*T*_3_	*T*_4_	*T*_5_
*Z*_1_	(0.75, 0.31, 0.37)	(0.59, 0.50, 0.55)	(0.59, 0.50, 0.55)	(0.75, 0.31, 0.37)	(0.75, 0.31, 0.37)
*Z*_2_	(0.37, 0.31, 0.75)	(0.59, 0.50, 0.55)	(0.59, 0.50, 0.55)	(0.75, 0.31, 0.37)	(0.59, 0.50, 0.55)
*Z*_3_	(0.59, 0.50, 0.55)	(0.59, 0.50, 0.55)	(0.59, 0.50, 0.55)	(0.59, 0.50, 0.55)	(0.75, 0.31, 0.37)
***H*_1_**	***T*** **_6_**	***T*** **_7_**	***T*** **_8_**	***T*** **_9_**	***T*** **_10_**
*Z*_1_	(0.75, 0.31, 0.37)	(0.59, 0.50, 0.55)	(0.59, 0.50, 0.55)	(0.59, 0.50, 0.55)	(0.37, 0.31, 0.75)
*Z*_2_	(0.75, 0.31, 0.37)	(0.75, 0.31, 0.37)	(0.59, 0.50, 0.55)	(0.37, 0.31, 0.75)	(0.59, 0.50, 0.55)
*Z*_3_	(0.59, 0.50,0.55)	(0.75, 0.31, 0.37)	(0.59, 0.50, 0.55)	(0.59, 0.50, 0.55)	(0.59, 0.50, 0.55)
***H*_1_**	***T*** **_11_**	***T*** **_12_**	***T*** **_13_**	***T*** **_14_**	***T*** **_15_**
*Z*_1_	(0.37, 0.31, 0.75)	(0.75, 0.31, 0.37)	(0.86, 0.23, 0.20)	(0.86, 0.23, 0.2)	(0.37, 0.31, 0.75)
*Z*_2_	(0.37, 0.31, 0.75)	(0.75, 0.31, 0.37)	(0.86, 0.23, 0.20)	(0.75, 0.31, 0.37)	(0.86, 0.23, 0.2)
*Z*_3_	(0.37, 0.31, 0.75)	(0.75, 0.31, 0.37)	(0.75, 0.31, 0.37)	(0.59, 0.50, 0.55)	(0.75, 0.31, 0.37)
***H*_1_**	***T*** **_16_**	***T*** **_17_**	***T*** **_18_**	***T*** **_19_**	***T*** **_20_**
*Z*_1_	(0.59, 0.50, 0.55)	(0.59 0.50, 0.55)	(0.37, 0.31, 0.75)	(0.37, 0.31, 0.75)	(0.75, 0.31, 0.37)
*Z*_2_	(0.75, 0.31, 0.37)	(0.75, 0.31, 0.37)	(0.86, 0.23, 0.20)	(0.86, 0.23, 0.20)	(0.75, 0.31, 0.37)
*Z*_3_	(0.86, 0.23, 0.20)	(0.86, 0.23, 0.20)	(0.75, 0.31, 0.37)	(0.86, 0.23, 0.20)	(0.75, 0.31, 0.37)
***H*_1_**	***T*** **_21_**	***T*** **_22_**	***T*** **_23_**	***T*** **_24_**	***T*** **_25_**
*Z*_1_	(0.75, 0.31, 0.37)	(0.59, 0.50, 0.55)	(0.75, 0.31, 0.37)	(0.37, 0.31, 0.75)	(0.75, 0.31, 0.37)
*Z*_2_	(0.75, 0.31, 0.37)	(0.75, 0.31, 0.37)	(0.75, 0.31, 0.37)	(0.75, 0.31, 0.37)	(0.75, 0.31, 0.37)
*Z*_3_	(0.75, 0.31, 0.37)	(0.75, 0.31, 0.37)	(0.75, 0.31, 0.37)	(0.75, 0.31, 0.37)	(0.75, 0.31, 0.37)

**Table A3:** The FSMD of expert *H*_2_.

*H*_2_	*T*_1_	*T*_2_	*T*_3_	*T*_4_	*T*_5_
*Z*_1_	(0.59, 0.50, 0.55)	(0.59, 0.50, 0.55)	(0.37, 0.31, 0.75)	(0.59, 0.50, 0.55)	(0.86, 0.23, 0.20)
*Z*_2_	(0.37, 0.31, 0.75)	(0.59, 0.50, 0.55)	(0.37, 0.31, 0.75)	(0.59, 0.50, 0.55)	(0.75, 0.31, 0.37)
*Z*_3_	(0.37, 0.31, 0.75)	(0.59, 0.50, 0.55)	(0.59, 0.50, 0.55)	(0.59, 0.50, 0.55)	(0.59, 0.50, 0.55)
***H*_2_**	***T*** **_6_**	***T*** **_7_**	***T*** **_8_**	***T*** **_9_**	***T*** **_10_**
*Z*_1_	(0.75, 0.31, 0.37)	(0.59, 0.5,0 0.55)	(0.75, 0.31, 0.37)	(0.59, 0.50, 0.55)	(0.59, 0.50, 0.55)
*Z*_2_	(0.37, 0.31, 0.75)	(0.75, 0.31, 0.37)	(0.75, 0.31, 0.37)	(0.59, 0.50, 0.55)	(0.59, 0.50, 0.55)
*Z*_3_	(0.75, 0.31, 0.37)	(0.75, 0.31, 0.37)	(0.75, 0.31, 0.37)	(0.59, 0.50, 0.55)	(0.59, 0.50, 0.55)
***H*_2_**	***T*** **_11_**	***T*** **_12_**	***T*** **_13_**	***T*** **_14_**	***T*** **_15_**
*Z*_1_	(0.37, 0.31, 0.75)	(0.75, 0.31, 0.37)	(0.59, 0.50, 0.55)	(0.86, 0.23, 0.20)	(0.75, 0.31, 0.37)
*Z*_2_	(0.37, 0.31, 0.75)	(0.75, 0.31, 0.37)	(0.75, 0.31, 0.37)	(0.86, 0.23, 0.20)	(0.75, 0.31, 0.37)
*Z*_3_	(0.37, 0.31, 0.75)	(0.86, 0.23, 0.20)	(0.86, 0.23, 0.20)	(0.86, 0.23, 0.20)	(0.75, 0.31, 0.37)
***H*_2_**	***T*** **_16_**	***T*** **_17_**	***T*** **_18_**	***T*** **_19_**	***T*** **_20_**
*Z*_1_	(0.75, 0.31, 0.37)	(0.59, 0.50, 0.55)	(0.37, 0.31, 0.75)	(0.37, 0.31, 0.75)	(0.75, 0.31, 0.37)
*Z*_2_	(0.75, 0.31, 0.37)	(0.75, 0.31, 0.37)	(0.86, 0.23, 0.20)	(0.86, 0.23, 0.20)	(0.86, 0.23, 0.20)
*Z*_3_	(0.75, 0.31, 0.37)	(0.86, 0.23, 0.20)	(0.75, 0.31, 0.37)	(0.86, 0.23, 0.20)	(0.75, 0.31, 0.37)
***H*_2_**	***T*** **_21_**	***T*** **_22_**	***T*** **_23_**	***T*** **_24_**	***T*** **_25_**
*Z*_1_	(0.75, 0.31, 0.37)	(0.75, 0.31, 0.37)	(0.75, 0.31, 0.37)	(0.59, 0.50, 0.55)	(0.75, 0.31, 0.37)
*Z*_2_	(0.75, 0.31, 0.37)	(0.75, 0.31, 0.37)	(0.75, 0.31, 0.37)	(0.75, 0.31, 0.37)	(0.75, 0.31, 0.37)
*Z*_3_	(0.75, 0.31, 0.37)	(0.75, 0.31, 0.37)	(0.86, 0.23, 0.20)	(0.75, 0.31, 0.37)	(0.75, 0.31, 0.37)

**Table A4:** The FSMD of expert *H*_3_.

*H*_3_	*T*_1_	*T*_2_	*T*_3_	*T*_4_	*T*_5_
*Z*_1_	(0.59, 0.50, 0.55)	(0.59 0.50, 0.55)	(0.75, 0.31, 0.37)	(0.75 0.31, 0.37)	(0.75, 0.31, 0.37)
*Z*_2_	(0.20, 0.12, 0.94)	(0.37 0.31, 0.75)	(0.37, 0.31, 0.75)	(0.75 0.31, 0.37)	(0.59, 0.50, 0.55)
*Z*_3_	(0.37, 0.31, 0.75)	(0.59 0.50, 0.55)	(0.37, 0.31, 0.75)	(0.75 0.31, 0.37)	(0.75, 0.31, 0.37)
***H*_3_**	***T*** **_6_**	***T*** **_7_**	***T*** **_8_**	***T*** **_9_**	***T*** **_10_**
*Z*_1_	(0.59, 0.50, 0.55)	(0.75 0.31, 0.37)	(0.37, 0.31, 0.75)	(0.59 0.50, 0.55)	(0.59, 0.50, 0.55)
*Z*_2_	(0.75, 0.31, 0.37)	(0.75 0.31, 0.37)	(0.37, 0.31, 0.75)	(0.37 0.31, 0.75)	(0.59, 0.50, 0.55)
*Z*_3_	(0.75, 0.31, 0.37)	(0.86 0.23, 0.20)	(0.59, 0.50, 0.55)	(0.37 0.31, 0.75)	(0.37, 0.31, 0.75)
***H*_3_**	***T*** **_11_**	***T*** **_12_**	***T*** **_13_**	***T*** **_14_**	***T*** **_15_**
*Z*_1_	(0.37, 0.31, 0.75)	(0.75 0.31, 0.37)	(0.75, 0.31, 0.37)	(0.75 0.31, 0.37)	(0.20, 0.12, 0.94)
*Z*_2_	(0.37, 0.31, 0.75)	(0.86 0.23, 0.20)	(0.86, 0.23, 0.20)	(0.75 0.31, 0.37)	(0.37, 0.31, 0.75)
*Z*_3_	(0.37, 0.31, 0.75)	(0.59 0.50, 0.55)	(0.37, 0.31, 0.75)	(0.37, 0.31, 0.75)	(0.86, 0.23 0.20)
***H*_3_**	***T*** **_16_**	***T*** **_17_**	***T*** **_18_**	***T*** **_19_**	***T*** **_20_**
*Z*_1_	(0.37, 0.31, 0.75)	(0.37 0.31, 0.75)	(0.59, 0.50, 0.55)	(0.59, 0.50, 0.55)	(0.59, 0.50, 0.55)
*Z*_2_	(0.59, 0.50, 0.55)	(0.75 0.31, 0.37)	(0.75, 0.31, 0.37)	(0.75, 0.31, 0.37)	(0.59, 0.50, 0.55)
*Z*_3_	(0.75, 0.31, 0.37)	(0.75 0.31, 0.37)	(0.75, 0.31, 0.37)	(0.75, 0.31, 0.37)	(0.86, 0.23, 0.20)
***H*_3_**	***T*** **_21_**	***T*** **_22_**	***T*** **_23_**	***T*** **_24_**	***T*** **_25_**
*Z*_1_	(0.75, 0.31, 0.37)	(0.75 0.31, 0.37)	(0.75, 0.31, 0.37)	(0.86, 0.23, 0.20)	(0.75, 0.31, 0.37)
*Z*_2_	(0.75, 0.31, 0.37)	(0.75 0.31, 0.37)	(0.75, 0.31, 0.37)	(0.75, 0.31, 0.37)	(0.75, 0.31, 0.37)
*Z*_3_	(0.75, 0.31. 0.37)	(0.75 0.31, 0.37)	(0.75, 0.31, 0.37)	(0.86, 0.23, 0.20)	(0.86, 0.23, 0.20)

**Table A5:** The normalized FSMD of expert *H*_1_.

*H*_1_	*T*_1_	*T*_2_	*T*_3_	*T*_4_	*T*_5_
*Z*_1_	(0.37, 0.31, 0.75)	(0.55, 0.50, 0.59)	(0.55, 0.50, 0.59)	(0.75, 0.31, 0.37)	(0.75, 0.31, 0.37)
*Z*_2_	(0.75, 0.31, 0.37)	(0.59, 0.50, 0.55)	(0.59, 0.50, 0.55)	(0.75, 0.31, 0.37)	(0.59, 0.50, 0.55)
*Z*_3_	(0.55, 0.50, 0.59)	(0.55, 0.50, 0.59)	(0.55, 0.50, 0.59)	(0.59, 0.50, 0.55)	(0.75, 0.31, 0.37)
***H*_1_**	***T*** **_6_**	***T*** **_7_**	***T*** **_8_**	***T*** **_9_**	***T*** **_10_**
*Z*_1_	(0.75, 0.31, 0.37)	(0.59, 0.50, 0.55)	(0.55, 0.50, 0.59)	(0.55, 0.50, 0.59)	(0.75, 0.31, 0.37)
*Z*_2_	(0.75, 0.31, 0.37)	(0.75, 0.31, 0.37)	(0.55, 0.50, 0.59)	(0.75, 0.31, 0.37)	(0.55, 0.50, 0.59)
*Z*_3_	(0.59, 0.50,0.55)	(0.75, 0.31, 0.37)	(0.55, 0.50, 0.59)	(0.55, 0.50, 0.59)	(0.55, 0.50, 0.59)
***H*_1_**	***T*** **_11_**	***T*** **_12_**	***T*** **_13_**	***T*** **_14_**	***T*** **_15_**
*Z*_1_	(0.75, 0.31, 0.37)	(0.75, 0.31, 0.37)	(0.86, 0.23, 0.20)	(0.86, 0.23, 0.2)	(0.37, 0.31, 0.75)
*Z*_2_	(0.75, 0.31, 0.37)	(0.75, 0.31, 0.37)	(0.86, 0.23, 0.20)	(0.75, 0.31, 0.37)	(0.86, 0.23, 0.2)
*Z*_3_	(0.75, 0.31, 0.37)	(0.75, 0.31, 0.37)	(0.75, 0.31, 0.37)	(0.59, 0.50, 0.55)	(0.75, 0.31, 0.37)
***H*_1_**	***T*** **_16_**	***T*** **_17_**	***T*** **_18_**	***T*** **_19_**	***T*** **_20_**
*Z*_1_	(0.59, 0.50, 0.55)	(0.59 0.50, 0.55)	(0.37, 0.31, 0.75)	(0.37, 0.31, 0.75)	(0.75, 0.31, 0.37)
*Z*_2_	(0.75, 0.31, 0.37)	(0.75, 0.31, 0.37)	(0.86, 0.23, 0.20)	(0.86, 0.23, 0.20)	(0.75, 0.31, 0.37)
*Z*_3_	(0.86, 0.23, 0.20)	(0.86, 0.23, 0.20)	(0.75, 0.31, 0.37)	(0.86, 0.23, 0.20)	(0.75, 0.31, 0.37)
***H*_1_**	***T*** **_21_**	***T*** **_22_**	***T*** **_23_**	***T*** **_24_**	***T*** **_25_**
*Z*_1_	(0.75, 0.31, 0.37)	(0.59, 0.50, 0.55)	(0.75, 0.31, 0.37)	(0.37, 0.31, 0.75)	(0.75, 0.31, 0.37)
*Z*_2_	(0.75, 0.31, 0.37)	(0.75, 0.31, 0.37)	(0.75, 0.31, 0.37)	(0.75, 0.31, 0.37)	(0.75, 0.31, 0.37)
*Z*_3_	(0.75, 0.31, 0.37)	(0.75, 0.31, 0.37)	(0.75, 0.31, 0.37)	(0.75, 0.31, 0.37)	(0.75, 0.31, 0.37)

**Table A6:** The normalized FSMD of expert *H*_2_.

*H*_2_	*T*_1_	*T*_2_	*T*_3_	*T*_4_	*T*_5_
*Z*_1_	(0.55, 0.50, 0.59)	(0.55, 0.50, 0.59)	(0.75, 0.31, 0.37)	(0.59, 0.50, 0.55)	(0.86, 0.23, 0.20)
*Z*_2_	(0.75, 0.31, 0.37)	(0.55, 0.50, 0.59)	(0.75, 0.31, 0.37)	(0.59, 0.50, 0.55)	(0.75, 0.31, 0.37)
*Z*_3_	(0.75, 0.31, 0.37)	(0.55, 0.50, 0.59)	(0.55, 0.50, 0.59)	(0.59, 0.50, 0.55)	(0.59, 0.50, 0.55)
***H*_2_**	***T*** **_6_**	***T*** **_7_**	***T*** **_8_**	***T*** **_9_**	***T*** **_10_**
*Z*_1_	(0.75, 0.31, 0.37)	(0.59, 0.5,0 0.55)	(0.37, 0.31, 0.75)	(0.55, 0.50, 0.59)	(0.55, 0.50, 0.59)
*Z*_2_	(0.37, 0.31, 0.75)	(0.75, 0.31, 0.37)	(0.37, 0.31, 0.75)	(0.55, 0.50, 0.59)	(0.55, 0.50, 0.59)
*Z*_3_	(0.75, 0.31, 0.37)	(0.75, 0.31, 0.37)	(0.37, 0.31, 0.75)	(0.55, 0.50, 0.59)	(0.55, 0.50, 0.59)
***H*_2_**	***T*** **_11_**	***T*** **_12_**	***T*** **_13_**	***T*** **_14_**	***T*** **_15_**
*Z*_1_	(0.75, 0.31, 0.37)	(0.75, 0.31, 0.37)	(0.59, 0.50, 0.55)	(0.86, 0.23, 0.20)	(0.75, 0.31, 0.37)
*Z*_2_	(0.75, 0.31, 0.37)	(0.75, 0.31, 0.37)	(0.75, 0.31, 0.37)	(0.86, 0.23, 0.20)	(0.75, 0.31, 0.37)
*Z*_3_	(0.75, 0.31, 0.37)	(0.86, 0.23, 0.20)	(0.86, 0.23, 0.20)	(0.86, 0.23, 0.20)	(0.75, 0.31, 0.37)
***H*_2_**	***T*** **_16_**	***T*** **_17_**	***T*** **_18_**	***T*** **_19_**	***T*** **_20_**
*Z*_1_	(0.75, 0.31, 0.37)	(0.59, 0.50, 0.55)	(0.37, 0.31, 0.75)	(0.37, 0.31, 0.75)	(0.75, 0.31, 0.37)
*Z*_2_	(0.75, 0.31, 0.37)	(0.75, 0.31, 0.37)	(0.86, 0.23, 0.20)	(0.86, 0.23, 0.20)	(0.86, 0.23, 0.20)
*Z*_3_	(0.75, 0.31, 0.37)	(0.86, 0.23, 0.20)	(0.75, 0.31, 0.37)	(0.86, 0.23, 0.20)	(0.75, 0.31, 0.37)
***H*_2_**	***T*** **_21_**	***T*** **_22_**	***T*** **_23_**	***T*** **_24_**	***T*** **_25_**
*Z*_1_	(0.75, 0.31, 0.37)	(0.75, 0.31, 0.37)	(0.75, 0.31, 0.37)	(0.59, 0.50, 0.55)	(0.75, 0.31, 0.37)
*Z*_2_	(0.75, 0.31, 0.37)	(0.75, 0.31, 0.37)	(0.75, 0.31, 0.37)	(0.75, 0.31, 0.37)	(0.75, 0.31, 0.37)
*Z*_3_	(0.75, 0.31, 0.37)	(0.75, 0.31, 0.37)	(0.86, 0.23, 0.20)	(0.75, 0.31, 0.37)	(0.75, 0.31, 0.37)

**Table A7:** The normalized SFDM of the expert *H*_3_.

*H*_3_	*T*_1_	*T*_2_	*T*_3_	*T*_4_	*T*_5_
*Z*_1_	(0.55, 0.50, 0.59)	(0.55 0.50, 0.59)	(0.37, 0.31, 0.75)	(0.75 0.31, 0.37)	(0.75, 0.31, 0.37)
*Z*_2_	(0.94, 0.12, 0.20)	(0.75 0.31, 0.37)	(0.75, 0.31, 0.37)	(0.75 0.31, 0.37)	(0.59, 0.50, 0.55)
*Z*_3_	(0.75, 0.31, 0.37)	(0.55 0.50, 0.59)	(0.75, 0.31, 0.37)	(0.75 0.31, 0.37)	(0.75, 0.31, 0.37)
***H*_3_**	***T*** **_6_**	***T*** **_7_**	***T*** **_8_**	***T*** **_9_**	***T*** **_10_**
*Z*_1_	(0.59, 0.50, 0.55)	(0.75 0.31, 0.37)	(0.75, 0.31, 0.37)	(0.55 0.50, 0.59)	(0.55, 0.50, 0.59)
*Z*_2_	(0.75, 0.31, 0.37)	(0.75 0.31, 0.37)	(0.75, 0.31, 0.37)	(0.75 0.31, 0.37)	(0.55, 0.50, 0.59)
*Z*_3_	(0.75, 0.31, 0.37)	(0.86 0.23, 0.20)	(0.55, 0.50, 0.59)	(0.75 0.31, 0.37)	(0.75, 0.31, 0.37)
***H*_3_**	***T*** **_11_**	***T*** **_12_**	***T*** **_13_**	***T*** **_14_**	***T*** **_15_**
*Z*_1_	(0.75, 0.31, 0.37)	(0.75 0.31, 0.37)	(0.75, 0.31, 0.37)	(0.75 0.31, 0.37)	(0.20, 0.12, 0.94)
*Z*_2_	(0.75, 0.31, 0.37)	(0.86 0.23, 0.20)	(0.86, 0.23, 0.20)	(0.75 0.31, 0.37)	(0.37, 0.31, 0.75)
*Z*_3_	(0.75, 0.31, 0.37)	(0.59 0.50, 0.55)	(0.37, 0.31, 0.75)	(0.37, 0.31, 0.75)	(0.86, 0.23 0.20)
***H*_3_**	***T*** **_16_**	***T*** **_17_**	***T*** **_18_**	***T*** **_19_**	***T*** **_20_**
*Z*_1_	(0.37, 0.31, 0.75)	(0.37 0.31, 0.75)	(0.59, 0.50, 0.55)	(0.59, 0.50, 0.55)	(0.59, 0.50, 0.55)
*Z*_2_	(0.59, 0.50, 0.55)	(0.75 0.31, 0.37)	(0.75, 0.31, 0.37)	(0.75, 0.31, 0.37)	(0.59, 0.50, 0.55)
*Z*_3_	(0.75, 0.31, 0.37)	(0.75 0.31, 0.37)	(0.75, 0.31, 0.37)	(0.75, 0.31, 0.37)	(0.86, 0.23, 0.20)
***H*_3_**	***T*** **_21_**	***T*** **_22_**	***T*** **_23_**	***T*** **_24_**	***T*** **_25_**
*Z*_1_	(0.75, 0.31, 0.37)	(0.75 0.31, 0.37)	(0.75, 0.31, 0.37)	(0.86, 0.23, 0.20)	(0.75, 0.31, 0.37)
*Z*_2_	(0.75, 0.31, 0.37)	(0.75 0.31, 0.37)	(0.75, 0.31, 0.37)	(0.75, 0.31, 0.37)	(0.75, 0.31, 0.37)
*Z*_3_	(0.75, 0.31. 0.37)	(0.75 0.31, 0.37)	(0.75, 0.31, 0.37)	(0.86, 0.23, 0.20)	(0.86, 0.23, 0.20)

**Table A8:** Aggregated spherical fuzzy decision matrix.

*H*	*T*_1_	*T*_2_	*T*_3_	*T*_4_
*Z*_1_	(0.5012, 0.4618, 0.6391)	(0.5500, 0.5000, 0.5900)	(0.5992, 0.3894, 0.5471)	(0.7073, 0.3731, 0.4223)
*Z*_2_	(0.8477, 0.2306, 0.3014)	(0.6348, 0.4338, 0.5050)	(0.6992, 0.3708, 0.4323)	(0.7073, 0.3731, 0.4223)
*Z*_3_	(0.6992, 0.3708, 0.4323)	(0.5500, 0.5000, 0.5900)	(0.6348, 0.4338, 0.5050)	(0.6552, 0.4360, 0.4819)
***H***	***T*** **_5_**	***T*** **_6_**	***T*** **_7_**	***T*** **_8_**
*Z*_1_	(0.7950, 0.2808, 0.3014)	(0.7073, 0.3731, 0.4223)	(0.6552, 0.4360, 0.4819)	(0.5992, 0.3894, 0.5471)
*Z*_2_	(0.6552, 0.4360, 0.4819)	(0.6718, 0.3187, 0.4683)	(0.7500, 0.3100, 0.3700)	(0.5992, 0.3894, 0.5471)
*Z*_3_	(0.7073, 0.3731, 0.4223)	(0.7073, 0.3731, 0.4223)	(0.7950, 0.2808, 0.3014)	(0.5012, 0.4618, 0.6391)
***H***	***T*** **_9_**	***T*** **_10_**	***T*** **_11_**	***T*** **_12_**
*Z*_1_	(0.5500, 0.5000, 0.5900)	(0.6348, 0.4338, 0.5050)	(0.7500, 0.3100, 0.3700)	(0.7500, 0.3100, 0.3700)
*Z*_2_	(0.6992, 0.3708, 0.4323)	(0.55,00 0.5000, 0.5900)	(0.75 0.31 0.37)	(0.7950, 0.2808, 0.3014)
*Z*_3_	(0.6348, 0.4338, 0.5050)	(0.6348, 0.4338, 0.5050)	(0.7500, 0.3100, 0.3700)	(0.7614, 0.3391, 0.3440)
***H***	***T*** **_13_**	***T*** **_14_**	***T*** **_15_**	***T*** **_16_**
*Z*_1_	(0.7614, 0.3391, 0.3440)	(0.8309, 0.2542, 0.2455)	(0.5357, 0.2917, 0.6389)	(0.6110, 0.3932, 0.5344)
*Z*_2_	(0.7950, 0.2808, 0.3014)	(0.7950, 0.2808, 0.3014)	(0.7338, 0.2878, 0.3814)	(0.7073, 0.3731, 0.4223)
*Z*_3_	(0.7338, 0.2878, 0.3814)	(0.6876, 0.3567, 0.4353)	(0.7950, 0.2808, 0.3014)	(0.7950, 0.2808, 0.3014)
***H***	***T*** **_17_**	***T*** **_18_**	***T*** **_19_**	***T*** **_20_**
*Z*_1_	(0.5331, 0.4662, 0.6099)	(0.4626, 0.4095, 0.6763)	(0.4626, 0.4095, 0.6763)	(0.7073, 0.3731, 0.4223)
*Z*_2_	(0.7500, 0.3100, 0.3700)	(0.8309, 0.2542, 0.2455)	(0.8309, 0.2542, 0.2455)	(0.7614, 0.3391, 0.3440)
*Z*_3_	(0.8309, 0.2542, 0.2455)	(0.7500, 0.3100, 0.3700)	(0.8309, 0.2542, 0.2455)	(0.7950, 0.2808, 0.3014)
***H***	***T*** **_21_**	***T*** **_22_**	***T*** **_23_**	***T*** **_24_**
*Z*_1_	(0.7500, 0.3100, 0.3700)	(0.7073, 0.3731, 0.4223)	(0.7500, 0.3100, 0.3700)	(0.6876 0.3567 0.4353)
*Z*_2_	(0.7500, 0.3100, 0.3700)	(0.7500, 0.3100, 0.3700)	(0.7500, 0.3100, 0.3700)	(0.7500, 0.3100, 0.3700)
*Z*_3_	(0.7500, 0.3100, 0.37)	(0.7500, 0.3100, 0.3700)	(0.7950, 0.2808, 0.3014)	(0.7950, 0.2808, 0.3014)
***H***	***T*** **_25_**			
*Z*_1_	(0.7500, 0.3100, 0.3700)			
*Z*_2_	(0.7500, 0.3100, 0.3700)			
*Z*_3_	(0.7950, 0.2808, 0.3014)			

**Table A9:** Aggregated weighted spherical fuzzy decision matrix.

	*T*_1_	*T*_2_	*T*_3_	*T*_4_
*Z*_1_	(0.1033, 0.0224, 0.9835)	(0.0546, 0.0057, 0.9956)	(0.1093, 0.0195, 0.9838)	(0.1382, 0.0249, 0.9763)
*Z*_2_	(0.2144, 0.0429, 0.9565)	(0.0653, 0.0066, 0.9944)	(0.1340, 0.0238, 0.9776)	(0.1382, 0.0249, 0.9763)
*Z*_3_	(0.1568, 0.0327, 0.9694)	(0.0546, 0.0057, 0.9956)	(0.1176, 0.0215, 0.9817)	(0.1244, 0.0231, 0.9799)
	***T*** **_5_**	***T*** **_6_**	***T*** **_7_**	***T*** **_8_**
*Z*_1_	(0.1872, 0.0371, 0.9581)	(0.1605, 0.0337 0.9681)	(0.1491, 0.0333, 0.9711)	(0.1097, 0.0197, 0.9837)
*Z*_2_	(0.1408, 0.0296, 0.9743)	(0.1494, 0.0307 0.9719)	(0.1806, 0.0383, 0.9609)	(0.1097, 0.0197, 0.9837)
*Z*_3_	(0.1564, 0.0320, 0.9697)	(0.1605, 0.0337 0.9681)	(0.1983, 0.0417, 0.953)	(0.0886, 0.0165, 0.9879)
	***T*** **_9_**	***T*** **_10_**	***T*** **_11_**	***T*** **_12_**
*Z*_1_	(0.0805, 0.0125, 0.9905)	(0.0833, 0.0107, 0.9908)	(0.1960, 0.0452 0.954)	(0.1980, 0.0462, 0.953)
*Z*_2_	(0.1097, 0.0159, 0.985)	(0.0696, 0.0093, 0.9929)	(0.1960, 0.0452 0.954)	(0.2173, 0.0503, 0.9436)
*Z*_3_	(0.0963, 0.0144, 0.9877)	(0.0833, 0.0107, 0.9908)	(0.1960, 0.0452 0.954)	(0.2027, 0.0478, 0.9497)
	***T*** **_13_**	***T*** **_14_**	***T*** **_15_**	***T*** **_16_**
*Z*_1_	(0.2084, 0.0506, 0.9468)	(0.2473, 0.0602, 0.9272)	(0.1387, 0.0349, 0.9746)	(0.1373, 0.0302 0.9748)
*Z*_2_	(0.2235, 0.0533, 0.9404)	(0.2289, 0.0560, 0.9375)	(0.2085, 0.0526, 0.9461)	(0.1669, 0.0365 0.9655)
*Z*_3_	(0.1971, 0.0470, 0.9518)	(0.1840, 0.0460, 0.9562)	(0.2363, 0.0597, 0.9334)	(0.1997, 0.0424 0.9523)
	***T*** **_17_**	***T*** **_18_**	***T*** **_19_**	***T*** **_20_**
*Z*_1_	(0.1199, 0.0282, 0.9788)	(0.1080, 0.0261, 0.9811)	(0.1144, 0.0293, 0.9788)	(0.1730, 0.0392, 0.9629)
*Z*_2_	(0.1876, 0.0414, 0.9578)	(0.2357, 0.0545, 0.9338)	(0.2492, 0.0611, 0.9261)	(0.1930, 0.0433, 0.9543)
*Z*_3_	(0.2226, 0.0485, 0.9410)	(0.1988, 0.0465, 0.9527)	(0.2492, 0.0611, 0.9261)	(0.2070, 0.0456, 0.9488)
	***T*** **_21_**	***T*** **_22_**	***T*** **_23_**	***T*** **_24_**
*Z*_1_	(0.1960, 0.0452 0.954)	(0.1698, 0.0378, 0.9643)	(0.2017, 0.0479, 0.9513)	(0.1740, 0.0410, 0.9609)
*Z*_2_	(0.1960, 0.0452 0.954)	(0.1851, 0.0403, 0.9589)	(0.2017, 0.0479, 0.9513)	(0.1973, 0.0458, 0.9534)
*Z*_3_	(0.1960, 0.0452 0.954)	(0.1851, 0.0403, 0.9589)	(0.2213, 0.0522, 0.9415)	(0.2165, 0.0499, 0.9440)
	***T*** **_25_**			
*Z*_1_	(0.2014, 0.0478, 0.9514)			
*Z*_2_	(0.2014, 0.0478, 0.9514)			
*Z*_3_	(0.2210, 0.0521, 0.9417)			

**Table A10:** The score of the SFNs.

Score	*T*_1_	*T*_2_	*T*_3_	*T*_4_	*T*_5_	*T*_6_	*T*_7_	*T*_8_	*T*_9_
*Z*_1_	−0.90721	−0.97426	−0.90524	−0.85929	−0.75612	−0.81229	−0.83176	−0.90447	−0.94484
*Z*_2_	−0.70834	−0.96596	−0.86696	−0.85929	−0.84947	−0.83451	−0.7699	−0.90447	−0.90982
*Z*_3_	−0.81983	−0.97426	−0.8924	−0.88142	−0.82152	−0.81229	−0.72783	−0.93116	−0.92714
**Score**	***T*** **_10_**	***T*** **_11_**	***T*** **_12_**	***T*** **_13_**	***T*** **_14_**	***T*** **_15_**	***T*** **_16_**	***T*** **_17_**	***T*** **_18_**
*Z*_1_	−0.94516	−0.73097	−0.72571	−0.69583	−0.58901	−0.84854	−0.85374	−0.87968	−0.89591
*Z*_2_	−0.95851	−0.73097	−0.67594	−0.65831	−0.6425	−0.69349	−0.79776	−0.75257	−0.62452
*Z*_3_	−0.94516	−0.73097	−0.7116	−0.72439	−0.75184	−0.62044	−0.72399	−0.66319	−0.72373
**Score**	***T*** **_19_**	***T*** **_20_**	***T*** **_21_**	***T*** **_22_**	***T*** **_23_**	***T*** **_24_**	***T*** **_25_**		
*Z*_1_	−0.88383	−0.78325	−0.73097	−0.79084	−0.71594	−0.777	−0.71666		
*Z*_2_	−0.58297	−0.73705	−0.73097	−0.75869	−0.71594	−0.72764	−0.71666		
*Z*_3_	−0.58297	−0.70441	−0.73097	−0.75869	−0.66449	−0.67821	−0.66533		

**Table A11:** The score accuracy degrees the SFNs.

Accuracy	*T*_1_	*T*_2_	*T*_3_	*T*_4_	*T*_5_	*T*_6_	*T*_7_	*T*_8_	*T*_9_
*Z*_1_	0.978501	0.994303	0.980262	0.972908	0.954389	0.964082	0.966471	0.980102	0.987768
*Z*_2_	0.962643	0.993044	0.974236	0.972908	0.969926	0.967773	0.957418	0.980102	0.982469
*Z*_3_	0.965319	0.994303	0.978063	0.976218	0.965801	0.964082	0.949316	0.984027	0.985109
**Accuracy**	***T*** **_10_**	***T*** **_11_**	***T*** **_12_**	***T*** **_13_**	***T*** **_14_**	***T*** **_15_**	***T*** **_16_**	***T*** **_17_**	***T*** **_18_**
*Z*_1_	0.988786	0.950534	0.949612	0.942399	0.924476	0.970288	0.970011	0.973229	0.974935
*Z*_2_	0.990801	0.950534	0.940173	0.937116	0.934396	0.941378	0.961385	0.954338	0.930553
*Z*_3_	0.988786	0.950534	0.945234	0.946996	0.950348	0.93064	0.948633	0.937292	0.949266
**Accuracy**	***T*** **_19_**	***T*** **_20_**	***T*** **_21_**	***T*** **_22_**	***T*** **_23_**	***T*** **_24_**	***T*** **_25_**		
*Z*_1_	0.972064	0.95871	0.950534	0.960107	0.947909	0.955193	0.948034		
*Z*_2_	0.923453	0.949852	0.950534	0.955423	0.947909	0.94995	0.948034		
*Z*_3_	0.923453	0.945163	0.950534	0.955423	0.938185	0.940568	0.93833		

**Table A12:** Strong concordance sets.

	*Z*_1_	*Z*_2_	*Z*_3_
*Z*_1_	{}	{}	{}
*Z*_2_	{}	{}	{}
*Z*_3_	{}	{}	{}

**Table A13:** Strong discordance sets.

	*Z*_1_	*Z*_2_	*Z*_3_
*Z*_1_	{}	{}	{}
*Z*_2_	{}	{}	{}
*Z*_3_	{}	{}	{}

**Table A14:** Distance measures for the computation of discordance indexes.

	*T*_1_	*T*_2_	*T*_3_	*T*_4_	*T*_5_	*T*_6_	*T*_7_	*T*_8_	*T*_9_
*d(Z*_1_, *Z*_1_)	0	0	0	0	0	0	0	0	0
*d(Z*_1_, *Z*_2_)	0.116138	0.010822	0.025895	0	0.049748	0.012088	0.033461	0	0.02995
*d(Z*_1_, *Z*_3_)	0.056304	0	0.008831	0.014411	0.033337	0	0.053022	0.021789	0.016106
*d(Z*_2_, *Z*_1_)	0.116138	0.010822	0.025895	0	0.049748	0.012088	0.033461	0	0.02995
*d(Z*_2_, *Z*_2_)	0	0	0	0	0	0	0	0	0
*d(Z*_2_, *Z*_3_)	0.059857	0.010822	0.017087	0.014411	0.016442	0.012088	0.019631	0.021789	0.013847
*d(Z*_3_, *Z*_1_)	0.056304	0	0.008831	0.014411	0.033337	0	0.053022	0.021789	0.016106
*d(Z*_3_, *Z*_2_)	0.059857	0.010822	0.017087	0.014411	0.016442	0.012088	0.019631	0.021789	0.013847
*d(Z*_3_, *Z*_3_)	0	0	0	0	0	0	0	0	0
	***T*_10_**	***T*** **_11_**	***T*** **_12_**	***T*** **_13_**	***T*** **_14_**	***T*** **_15_**	***T*** **_16_**	***T*** **_17_**	***T*** **_18_**
*d(Z*_1_, *Z*_1_)	0	0	0	0	0	0	0	0	0
*d(Z*_1_, *Z*_2_)	0.013866	0.02153	0.077376	0.031667	0.072051	0.139109	0.148282	0.022154	0
*d(Z*_1_, *Z*_3_)	0	0.071044	0.10875	0.067461	0.111271	0.09725	0.148282	0.037345	0
*d(Z*_2_, *Z*_1_)	0.013866	0.02153	0.077376	0.031667	0.072051	0.139109	0.148282	0.022154	0
*d(Z*_2_, *Z*_2_)	0	0	0	0	0	0	0	0	0
*d(Z*_2_, *Z*_3_)	0.013866	0.049607	0.031393	0.035852	0.039493	0.042265	0	0.0152	0
*d(Z*_3_, *Z*_1_)	0	0.071044	0.10875	0.067461	0.111271	0.09725	0.148282	0.037345	0
*d(Z*_3_, *Z*_2_)	0.013866	0.049607	0.031393	0.035852	0.039493	0.042265	0	0.0152	0
*d(Z*_3_, *Z*_3_)	0	0	0	0	0	0	0	0	0
	***T*** **_19_**	***T*** **_20_**	***T*_21_**	***T*_22_**	***T*_23_**	***T*** **_24_**	***T*** **_25_**		
*d(Z*_1_, *Z*_1_)	0	0	0	0	0	0	0		
*d(Z*_1_, *Z*_2_)	0.016405	0	0	0.021848	0.016559	0.024922	0		
*d(Z*_1_, *Z*_3_)	0.016405	0.022326	0	0.005943	0.012922	0.046562	0.022291		
*d(Z*_2_, *Z*_1_)	0.016405	0	0	0.021848	0.016559	0.024922	0		
*d(Z*_2_, *Z*_2_)	0	0	0	0	0	0	0		
*d(Z*_2_, *Z*_3_)	0	0.022326	0	0.016046	0.029426	0.021753	0.022291		
*d(Z*_3_, *Z*_1_)	0.016405	0.022326	0	0.005943	0.012922	0.046562	0.022291		
*d(Z*_3_, *Z*_2_)	0	0.022326	0	0.016046	0.029426	0.021753	0.022291		
*d(Z*_3_, *Z*_3_)	0	0	0	0	0	0	0		
